# The *FIBRILLIN* multigene family in tomato, their roles in plastoglobuli structure and metabolism

**DOI:** 10.1111/tpj.70447

**Published:** 2025-09-09

**Authors:** Juliana Almeida, Laura Perez‐Fons, Margit Drapal, Kit Liew, Eugenia M. A. Enfissi, Paul D. Fraser

**Affiliations:** ^1^ Department of Biological Sciences Royal Holloway University of London Egham Surrey TW20 0EX UK; ^2^ Present address: LongPing High‐Tech Rodovia SP 330, 14 140‐000 Cravinhos Brazil; ^3^ Present address: Division of Biological and Environmental Sciences and Engineering 4700 King Abdullah University of Science and Technology Thuwal 23955‐6900 Kingdom of Saudi Arabia

**Keywords:** plastoglobuli, carotenoids, isoprenoids, chromoplast, esterification, lipid homeostasis, fibrillin, multiplexing gene editing

## Abstract

Plastoglobuli (PG) are plant lipoprotein compartments, present in plastid organelles. They are involved in the formation and/or storage of lipophilic metabolites. FIBRILLINs (FBNs) are one of the main PG‐associated proteins and are particularly abundant in carotenoid‐enriched chromoplasts found in ripe fruits and flowers. To address the contribution of different FBNs, independently and in combination, to isoprenoid formation and sequestration, a multiplex gene editing approach was undertaken in tomato. This approach generated a suite of single and high‐order *fbn* mutants that were shown to lack transcripts and respective protein products. The major PG‐related FBNs in tomato chosen for this study are *Sl*FBN1, *Sl*FBN2a, *Sl*FBN4 and *Sl*FBN7a. When knocked out independently, functional redundancy was revealed. However, paralog‐specific roles were detected regulating specific isoprenoids (e.g. plastochromanol 8) or plastidial esterification capability. In addition, high‐order *fbn* mutants displayed altered isoprenoid chromoplast sequestration patterns, notably with a significant reduction in carotenes (phytoene and phytofluene) in the PG fraction. Proteomic analysis confirmed the absence of PG‐core associated proteins, including NAD(P)H‐ubiquinone oxidoreductase C1, tocopherol cyclase (VTE1) and phytol esterase (PES1/PYP). Perturbations to the ultrastructure of the plastid were revealed, with aberrant PG formation and morphology predominating in high‐order mutants. Global lipidome profiles also highlighted broader changes directly affecting storage and plastid membrane lipids, for example, tri‐ and diacylglycerides and galactolipid species. Collectively, these results support both structural and metabolic roles of *Sl*FBNs in PGs. The findings expose fundamental aspects of metabolic compartmentalisation in plant cells and the importance of lipoprotein particles for plastid integrity and functionality.

## Significance Statement

A multiplex CRISPR/Cas approach has been used to characterise members of the FIBRILLIN (FBN) multigene family, through the generation and characterisation of independent and high‐order mutants. Molecular and biochemical characterisation has been performed, showing functional redundancy between members and the roles of FBN in plastoglobuli (PG) biogenesis and lipid metabolism.

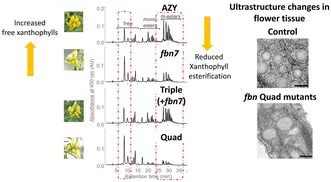

A multiplex gene editing methodology has been used to generate independent and higher order mutants for the FBN gene family. Characterisation of these valuable resources has revealed an involvement of FBNs in PG biogenesis, lipid remodelling and isoprenoid formation. This genetic resource will now aid the elucidation of the role of FBNs and PG in carotenoid‐enriched tissues, with potential applications in improving crops for nutritional quality and resilience to abiotic stresses associated with climate change.

## INTRODUCTION

The role of lipid droplets and lipoproteins in maintaining lipid homeostasis has been studied extensively in animal systems due to their involvement in cardiovascular diseases and obesity (Goldstein & Brown, 2009). Similar lipoprotein structures are also present in the plastids of plants. However, their function remains comparatively poorly understood (Lundquist et al., 2020). Plant plastids, including photosynthetically active chloroplasts and other types, contain dynamic lipid storage sub‐compartments termed plastoglobuli (PGs). PG number, size and composition vary depending on plastid differentiation and type, being linked to plant developmental transitions. PG characteristics can also change as a result of stress responses (Lichtenthaler, 2013; van Wijk & Kessler, 2017). Recent evidence has supported that the PG is not only a sink for lipids but also an active metabolic hub containing key metabolites of biotechnological importance (Rottet et al., 2015; Spicher & Kessler, 2015).

Structurally, PGs are surrounded by a membrane lipid monolayer, encapsulating a neutral lipid core. Triacylglycerides (TAGs) can predominate in the core, depending on plastid type, but also present are isoprenoids (carotenoids, tocopherols and other prenylquinones) (Bréhélin et al., 2007). A specific set of proteins coat the PG surface, which also greatly vary depending on cell type (van Wijk & Kessler, 2017). A major structural protein class found in PGs is FIBRILLINs (FBNs). These plastid‐specific proteins are encoded by a multigene family whose members contain a typical plastid lipid‐associated protein (PAP) domain. They do, however, lack integral transmembrane domains but possess amphipathic helices predicted to bind to the PG lipid monolayer (Shanmugabalaji et al., 2013; Singh & McNellis, 2011). FBNs also contain a conserved lipocalin‐like motif which has been suggested to participate in the binding and transport of small hydrophobic molecules (Kim et al., 2017; Singh & McNellis, 2011).

In the model flowering plant Arabidopsis, 14 FBNs have been identified; seven of which are considered PG‐associated proteins, while the others are located primarily in thylakoid membranes or stroma of chloroplasts (Lundquist et al., 2012; van Wijk & Kessler, 2017). Functionally, FBNs are involved in different plant processes ranging from photosynthesis to colour acquisition during organ development and response to stress conditions (Langenkämper et al., 2001; Leitner‐Dagan et al., 2006; Youssef et al., 2010). Moreover, FBNs can fulfil key structural roles associated with metabolic pathways (Kim et al., 2015, 2017).

FBNs are best known for their role in flower and fruit chromoplasts, where they are involved in metabolism for the synthesis and storage of carotenoids and isoprenoid‐derived compounds with high nutritional and industrial value (Nogueira et al., 2018; Sun et al., 2020). FBN1/PAP and its homologues in different species are required for the formation of carotenoid‐sequestering structures occurring during fruit ripening such as PGs and fibrils (Deruere et al., 1994; Fang et al., 2020; Kilambi et al., 2013; Leitner‐Dagan et al., 2006; Suzuki et al., 2015). Enhanced‐carotenoid plant genotypes produced by biotechnological interventions typically exhibit altered chromoplast structure with increased PG number, highlighting the metabolic composition influence on plastid morphology (Berry et al., 2019; Enfissi et al., 2010, 2019; Nogueira et al., 2013). Despite multiple studies implicating FBNs with PG formation and dynamics, the underlying mechanisms of how FBNs fulfil this role remain poorly understood. It has been proposed that PG‐targeted FBNs may be involved in globule size, preventing PG coalescence and favouring their clustering (Rey et al., 2000). Given the multigene feature of FBNs, questions remain as to which FBN members act on PG formation, and/or which affect PG core lipid remodelling, leading to changes in plant metabolism. Indeed, individual FBN contributions are challenging to interpret because several members occur simultaneously in plant cells.

In the present study, the independent and complementary roles of the major PG‐related FBNs have been investigated by adopting a multiplexing gene editing approach in tomato. Metabolomic, proteomic, and cellular ultrastructural analysis has provided a deeper insight into the functional diversity of the *fbn* gene family and how they can be exploited in future biotechnological applications.

## RESULTS

### Putative plastoglobular FBNs associated with tomato chromoplast development

**Figure 1:**
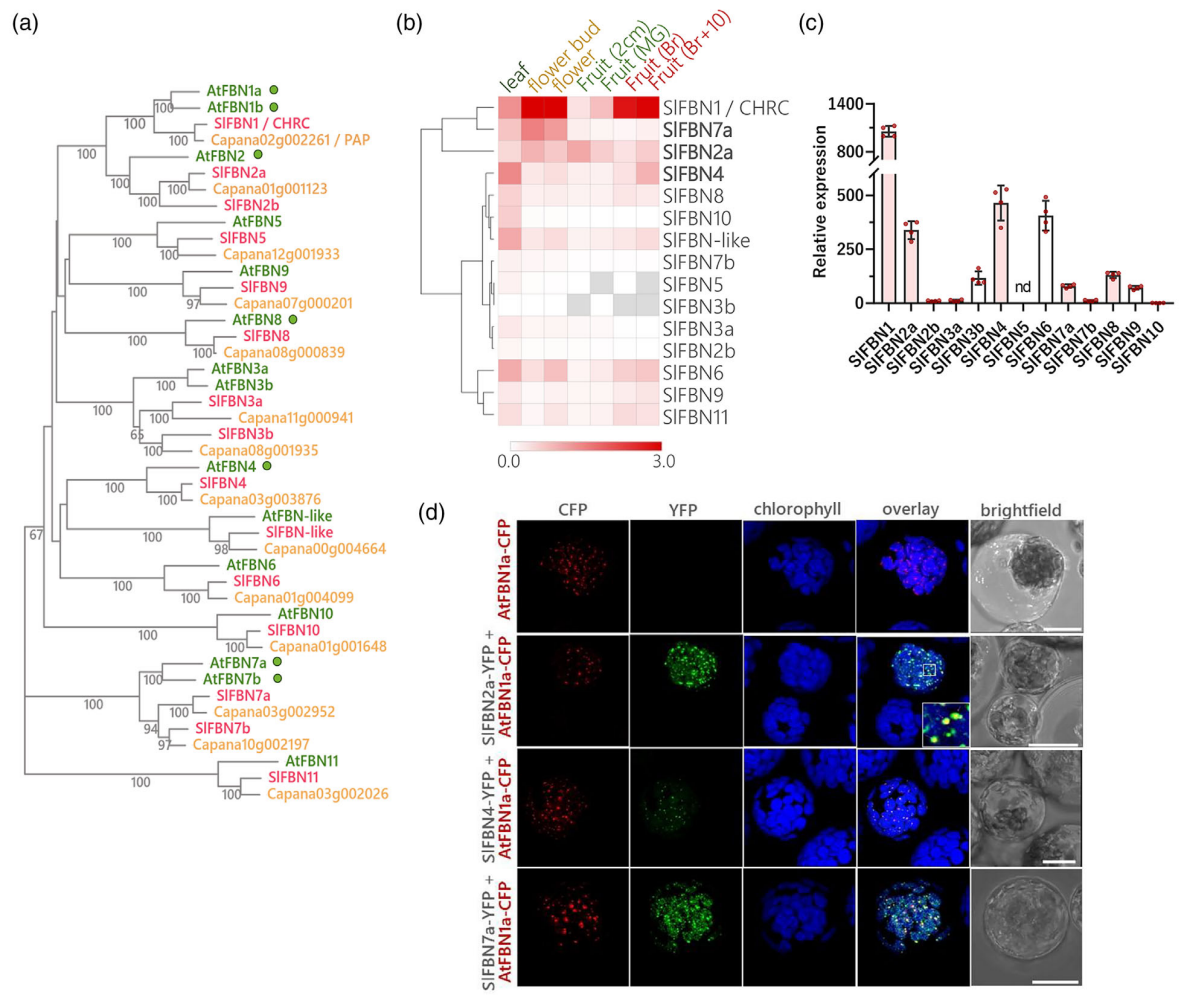
Identification of tomato FIBRILLINs (*Sl*FBNs), expression profile and subcellular localisation. (a) Phylogenetic analysis of FBN proteins from *Arabidopsis thaliana* (*At*FBN), *Solanum lycopersicum* (*Sl*FBN), *Capsicum annuum* (cv. Zunla) inferred using the Neighbour‐Joining method with 1000 bootstrap replicates. Numbers in the nodes denote bootstrap confidence levels (only values >50 are shown). Green circles indicate plastoglobuli (PG)‐related *At*FBNs (van Wijk & Kessler, 2017). *Solanum lycopersicum* gene IDs are listed in Table S9. (b) Heatmap representation of *Slfbn* expression profile. Normalised expression levels were obtained from the TomExpress database (http://tomexpress.toulouse.inra.fr). (c) Relative expression of *Slfbn* genes in fruits at 1 day post‐breaker (B1) stage by quantitative polymerase chain reaction. Values are expression levels normalised to *cac* and *act2* reference genes (mean ± SEM, *n* = 4 biological replicates). Tomato orthologues of PG‐related FBNs were highlighted in red. Nd, not detected. (d) Transient co‐expression of full‐length *Sl*FBN tagged to YFP (*Sl*FBN2a‐YFP, *Sl*FBN4‐YFP and *Sl*FBN7a‐YFP), with PG marker (*At*FBN1a‐CFP) into protoplasts isolated from Arabidopsis (Ler0) leaves. The CFP, YFP, chlorophyll and merged fluorescence, obtained from a confocal microscope, are shown in columns of the panel from the left to the right. Bright field images of protoplasts were shown in the last column. Co‐expression of all *Sl*FBN‐YFP and *At*FBN1a‐CFP led, at least, to partial co‐localisation. Scale bars = 20 μm.

Using the *Arabidopsis thaliana* FBN (*At*FBN) protein sequences (van Wijk & Kessler, 2017), 15 tomato *fbn* (*Slfbn*) genes were identified in total covering all *At*FBN groups. For comparison, homologous FBN sequences from pepper (*Capsicum annuum*, *CaFBN*s), another Solanaceae species with fruits highly specialised for carotenoid storage, were also retrieved. *Slfbn* orthologs of *Atfbn*s encoding PG‐targeted proteins (Lundquist et al., 2012), namely *Slfbn1/chrc, ‐2a, ‐2b, ‐4, ‐7a, ‐7b, ‐8* were determined by phylogenetic analysis. Notably, paralogous pairs primarily clustered within each plant family, supporting lineage‐specific expansions (Figure 1a).

Of the seven PG‐related *Slfbn* transcripts, levels of five members (*Slfbn1*, ‐*2a*, ‐*7a*, ‐*4*, ‐*8*) were highest at carotenoid‐enriched stages of flower and fruit development, based on transcriptome database queries (Figure 1b). Their expression profile was further confirmed by quantitative polymerase chain reaction (qPCR) analysis in fruit (Figure 1c). *Slfbn1/chrc* was confirmed as the most highly chromoplast‐expressed *Slfbn* (Kilambi et al., 2013) and correlated to *Slfbn2a* and ‐*7a* expression (Figure 1b). Besides, *Slfbn4* transcripts also accumulated as tomato fruit ripening advanced. The qualitative expression profiles of the poorly characterised *Slfbn6* transcripts were observed to be similar to the characterised *Slfbn1* transcripts and, therefore, for logistical priorities, the *Slfbn1* was included for the functional characterisation of higher order mutants.

Pepper transcriptomic data (Liu et al., 2017) was also interrogated to check *Cafbn* transcript abundance across fruit development. Interestingly, PG‐related *Cafbn* homologues had expression patterns similar to tomato (highly expressed at chromoplast‐enriched tissues/stages) (Figure S1). *Cafbn1/pap* was the predominant *Cafbn* expressed in fruits. Despite differences in flowers due to the lack of chromoplasts in pepper petals, high expression rates of *Cafbn4*, *Cafbn2* and *Cafbn7a* were detected at the late stages of pepper fruit ripening, arguing for their role in carotenoid sequestration.

Based on the above orthologues and expression profiles, *Sl*FBN2a, *Sl*FBN4 and *Sl*FBN7a emerged as candidates involved in PG biogenesis and carotenoid sequestration during fruit ripening, potentially complementing *Sl*FBN1/CHRC function in chromoplasts. To first check their subcellular localisation, each *Sl*FBN candidate, with a yellow fluorescent protein tag (*Sl*FBN‐YFP), was transiently co‐expressed with the plastoglobular marker *At*FBN1a (Vidi et al., 2006) in protoplasts. The signal fluorescence of all *Sl*FBN‐YFP fusions was restricted to the plastids. While *Sl*FBN4‐YFP fluorescence greatly co‐localised with the PG marker, the patterns of *Sl*FBN2a‐YFP and *Sl*FBN7a‐YFP suggested a more diffuse distribution of these proteins within the plastid, not restricted to PG (Figure 1d). Together, the results suggest that *Sl*FBN2a, ‐4, ‐7a can be associated with PGs and likely have a role in PG formation and composition.

### Generation of single and high‐order *fbn* mutants by CRISPR‐Cas

**Figure 2:**
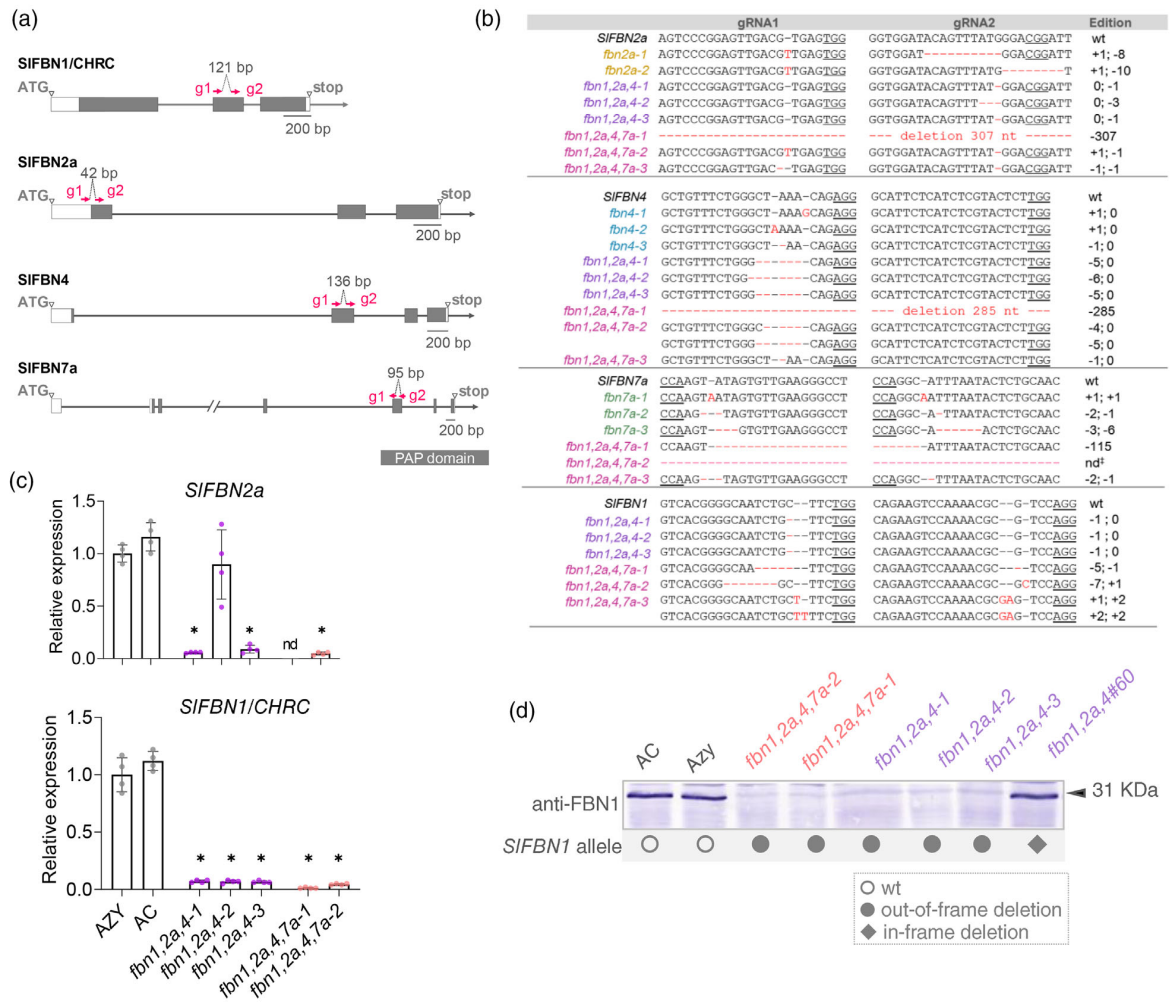
Gene‐edited *fbn* mutants generated by CRISPR‐Cas. (a) Intron/exon structure of *Slfbn1*, *Slfbn2a*, *Slfbn4*, *Slfbn7a* locus. PAP/FIBRILLIN domain is indicated in grey. (b) Nucleotide sequences showing induced mutations in *Slfbn* inherited mutations in T_2_ transgene null‐segregant plants. Indels are highlighted in red characters. (c) Relative expression of *Slfbn1/chrc* and *Slfbn2a* in ripe fruits (B7, 7 days post‐breaker) by quantitative polymerase chain reaction. Values are relative expression levels (mean ± SD, *n* ≥ 3 biological replicates) to Azy control. Asterisks indicate statistically significant differences (one‐way anova, Dunnett's multiple post‐test, *P < 0.05, compared to Azy). (d) Immunoblotting analysis of *Sl*FBN1. Proteins extracted from B7 ripe fruits (10 μg) were separated on SDS/PAGE, blotted and membranes probed for the presence of the FBN1 with antiserum raised against *Capsicum* FBN1/PAP. An extra T_1_ *Cas* ^+^ line (*fbn1,2a,4*#60) harbouring in‐frame indel was included in the analysis for comparison.

To address the contribution of *Sl*FBN2a, ‐4, ‐7a in PG formation and isoprenoid sequestration, single loss‐of‐function mutants for the candidate *Sl*FBNs were generated via CRISPR‐Cas technology (Figure 2; Table S1). To assess functional redundancy within the FBN family, a multiplexing approach was also undertaken to simultaneously deliver combinations of mutant alleles for *Sl*FBN1, ‐2a, ‐4 (triple mutants) and *Sl*FBN1, ‐2a, ‐4, ‐7a (quadruple mutants) (Figure S2).

Sequencing of the targeted genomic regions confirmed edited alleles in primary T_0_ transformants obtained through single and multiplexing strategies. Editing efficiency ranged from 67 to 100% (Table S2). Mutations occurred in different combinations: chimera, biallelic and homozygous, being mostly short indels upstream of PAM sites. Large out‐of‐frame deletions (>100 bp) were also detected at a lower frequency. Edited alleles harbouring frameshift or nonsense (premature stop codon, PTC) mutations were predicted as knock‐outs. Lines carrying in‐frame deletion alleles, which could either retain functionality or be loss‐of‐function, were also selected for comparison when available. Possible off‐target sites for the gRNAs examined in T_0_ and T_1_ plants (Table S1) were undetected, supporting the high specificity of the gRNAs designed. T_2_ Cas‐free homozygous/biallelic *Slfbn* plants were successfully established and used for further comprehensive phenotyping and molecular characterisation (Figure 2; Table S3). Since transcripts with PTC introduced by gene editing can be targeted by nonsense‐mediated decay pathways, by which nonsense mRNAs are destroyed (Popp & Maquat, 2016), expression of *Slfbn1*/*chrc* and *Slfbn2a*, both highly expressed in fruits, was checked by qPCR. Levels of PTC‐containing mutant transcripts were reduced in *fbn* fruits compared to their control (Figure 2c). Additionally, FBN1 protein levels were checked by immunoblotting using an antibody to *Ca*FBN1/PAP (Deruere et al., 1994). *Sl*FBN1 was exclusively detected in fruits from edited lines carrying either wild‐type or in‐frame deletion alleles (Figure 2d).

Visual inspection of *fbn* mutants revealed no obvious difference in fruit colour but altered flower pigmentation. The typical bright yellow colour failed to develop, particularly in *fbn* mutants harbouring *Slfbn7a* defective alleles (single *fbn7a* and quadruple *fbn1,2a,4,7a*) (Figure 5a). Triple and quadruple mutants, under non‐stressed growth conditions, had some altered plant morphological traits. These morphological changes consisted of a shorter internode length compared to their controls, and visually, leaf areas were reduced in the quadruple mutants (Figure S3b). No significant changes were detected in maximum quantum efficiency of PSII (Fv/Fm) of *fbn* mutants, except for the *fbn1,2a,4,7a‐1* quadruple mutant (Figure S3d).

### *Sl*FBN homologues influence the accumulation of carotenoids and other isoprenoids in plastids

**Figure 3:**
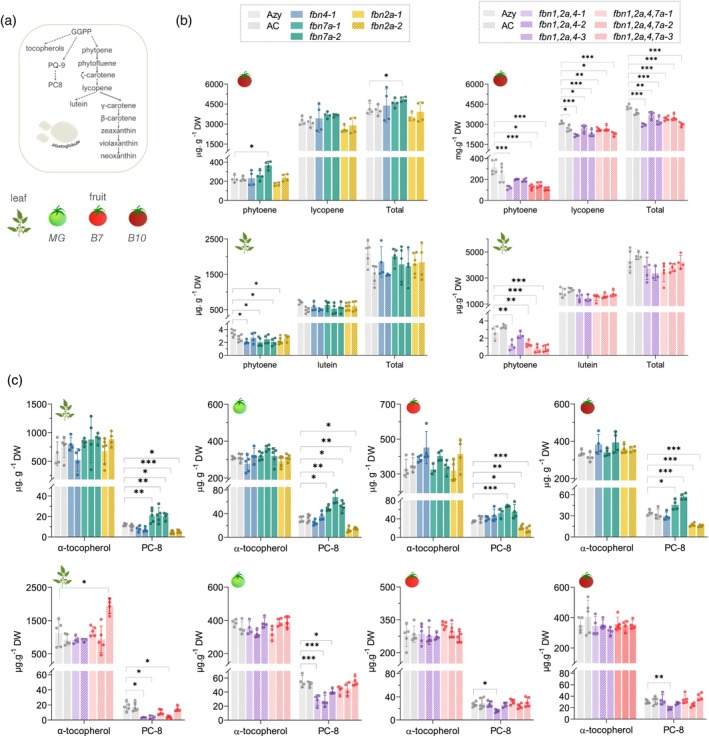
*Sl*FBNs altered isoprenoid profile in tomato fruits and leaves of *fbn* mutants. (a) Schematic representation of the plastidial biosynthetic pathway of isoprenoid compounds determined by ultra‐performance liquid chromatography‐photo diode array and the corresponding sample set: fruits at mature green, ripe (B7 and B10) stages and leaves. (b) Scatter‐plot showing representative carotenoids in ripe fruit (B10) and leaves. Each dot represents a single biological replicate. Data (*n* ≥ 4, biological replicates) were analysed by one‐way anova with Dunnett's post‐tests; asterisks denote statistically significant differences (*P < 0.05, **P < 0.01, ***P < 0.001) compared to azygous (Azy) control. Error bars indicate ±SD. (c) α‐tocopherol and PC‐8 levels. AC, wild‐type; GGPP, geranylgeranyl pyrophosphate; PC‐8, plastochromanol; PQ‐9, plastoquinone. Full data set available in Table S4.

To assess how *Slfbn* mutations influence plastidial isoprenoid metabolism, fruit and leaf isoprenoid profiles were first examined by ultra‐performance liquid chromatography‐photo diode array (UPLC‐PDA) (Figure 3; Table S4). Three fruit stages were analysed: mature green (MG), red ripe (B7) and late red ripe (B10). No significant changes in carotenoids or chlorophylls in leaf or MG tissues were observed (Table S4), except foliar phytoene. Carotenoid changes occurred predominantly in ripe fruits from high‐order *fbn* mutants (Figure 3b; Table S4). The early carotene intermediates (phytoene, phytofluene and ζ‐carotene) were most affected and reduced by 60% in the mutants compared to control (Azygous, Azy) at the B10 stage. Unexpectedly, the decrease in lycopene, the main carotenoid accumulating in red fruit, was less pronounced (levels of 80% retained). β‐Carotene levels, however, were unaffected despite lower levels of its immediate precursor γ‐carotene. In single *fbn* fruits, carotenoid content remained similar to controls at all fruit stages.

Other isoprenoids known to be sequestered in the PG core, such as tocopherol and quinone derivatives, responded differently to single or multiple losses of *Sl*FBN*s* (Figure 3b; Table S4). Despite similar levels of α‐tocopherol and plastoquinone (PQ‐9), plastochromanol‐8 (PC‐8) significantly changed in *fbn* mutants. PC‐8 is a PQ‐9 derivative predominantly found in PG associated with stress responses (Zbierzak et al., 2010). While the lack of *Sl*FBN2a approximately halved PC‐8 levels compared to the control, deficiency in *Sl*FBN7a had an opposite effect, favouring PC‐8 accumulation with up to a twofold increase. Of note, similar PC‐8 levels observed among *fbn7a* lines carrying either in‐frame (*fbn7a‐3*) or nonsense alleles (*fbn7a‐1*, *fbn7a‐2*) suggested that all *Slfbn7a* editions likely derived from *Slfbn7a* null alleles. In high‐order *fbn* mutants, *Slfbn2a* and *Slfbn7a* defective alleles displayed compensatory interaction to modulate PC‐8 levels, though outcomes were specific to the organ and developmental stages. In triple mutants, PC‐8 levels were decreased in chloroplast‐containing tissues, MG fruits (50%) and leaves (20%), compared to the control, while no consistent effect was found in red fruits. In quadruple mutants, the addition of a defective *Sl*FBN7a restored PC‐8 levels in fruits at all stages. Together, these findings define mutation‐specific effects of *Sl*FBNs on levels of prenylquinones. While *Sl*FBN2a favours PC‐8 accumulation, *Sl*FBN7a prevents it. To further substantiate these findings, an extra set of T_1_ *Slfbn*‐edited lines harbouring Cas9‐gRNA transgene (*Cas* ^+^) with the following genotypes: triple (*Cas* ^+^ */fbn1,2a,4*), quadruple (*Cas* ^+^ */fbn1,2a,4,7a*) and double (*Cas* ^+^ */fbn2a,4*) mutant (Table S3) were analysed. This additional set of *fbn* mutants allowed us not only to confirm the fruit profile found in T_2_ Cas‐free progeny but also to evaluate *Sl*FBN1‐specific contribution to the isoprenoid profile by comparing *Cas* ^+^ */fbn2a,4* and *Cas* ^+^ */fbn1,2a,4* (Table S4). PC‐8 levels were similar between double and triple mutants, implicating *Sl*FBN2a as the major positive regulator of PC‐8 levels. The levels of PQ‐9 were also analysed in the tissue to complement the determinations from sub‐plastidial fractions and no change was observed (Table S4).

### Changes in isoprenoid sequestration in fruit chromoplasts of *fbn* mutants

**Figure 4:**
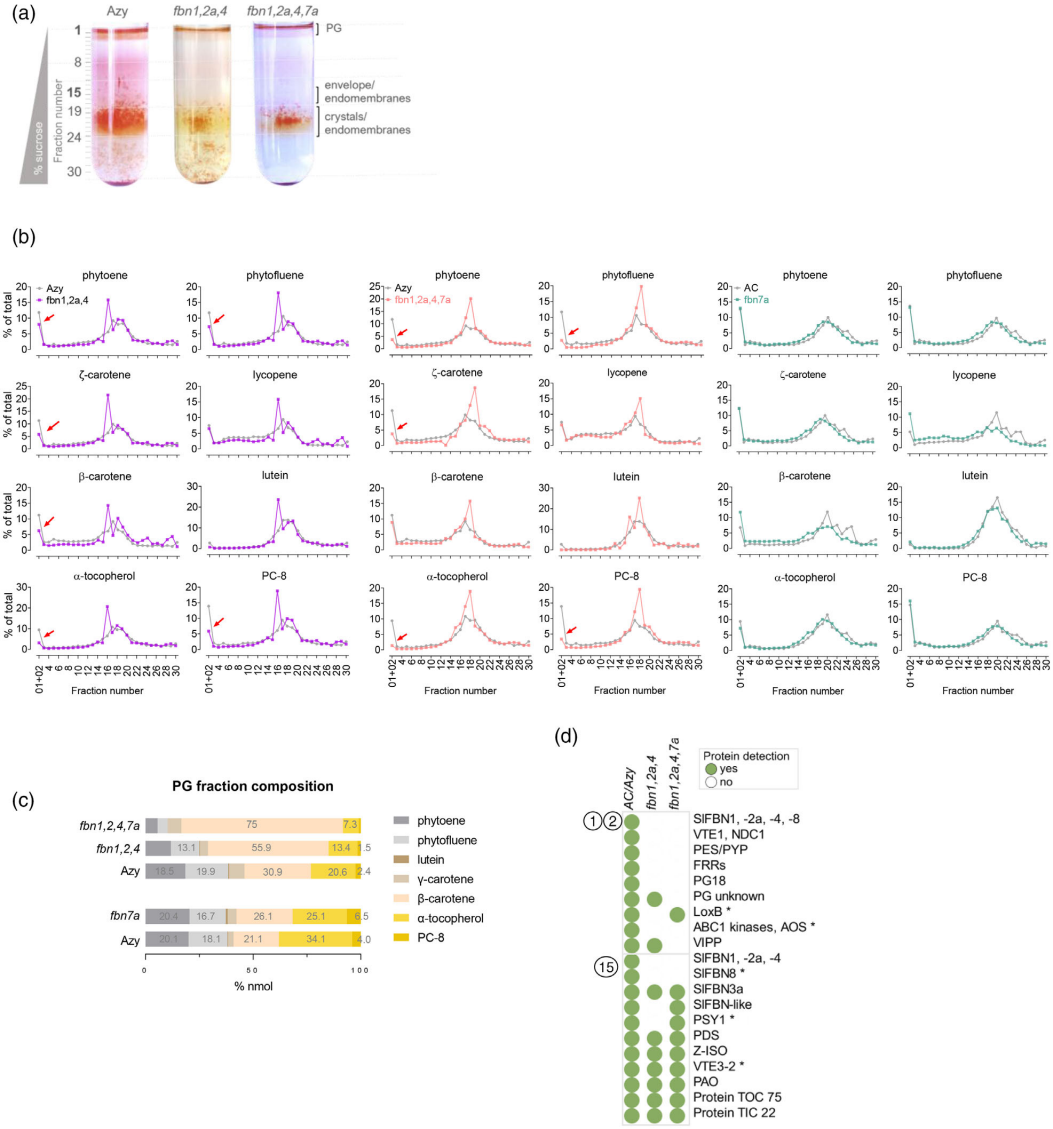
Sequestration of isoprenoids into sub‐chromoplast compartments in *fbn* mutants. (a) Separation of membranes from isolated tomato chromoplasts (fruits at 3–5 days post breaker stage) by flotation on a discontinuous sucrose gradient. Plastoglobuli (PG)‐enriched buoyant fractions (F1+F2) form a red layer at the top of the gradient. (b) Isoprenoid profile of sub‐chromoplast fractions obtained from triple (*fbn1,2a,4*), quadruple (*fbn1,2a,4,7a*) and *fbn7a* mutants. Values represent % of the total found in each fraction. Red arrows indicate reduced isoprenoid sequestration into PG sub‐compartment. (c) Isoprenoid composition (% nmol) of PG fractions; for this comparison, lycopene was excluded from analysis. (d) Representative proteins found in PG‐enriched (F1+F2) and top membrane/envelope‐enriched (F15) fractions based on proteomic analysis. Proteins were isolated from fractions, separated by SDS‐PAGE; excised gel fragments were trypsinised, and the resulting peptides analysed by mass spectrometry. Asterisks denote proteins found only in one of the control lines (AC or Azy).

*Sl*FBN‐dependent accumulation of isoprenoids may be linked to how these compounds are preferentially sequestered across plastid sub‐compartments (Nogueira et al., 2013). To test this hypothesis, sub‐plastidial chromoplast fractionations were isolated and analysed for isoprenoid contents (Figure 4). A differential sequestration pattern was observed in high‐order *fbn* mutants (Figure 4b) compared to the *fbn*7a line and their controls. For example, the proportion of phytoene and phytofluene was decreased in the PG fraction of higher order *fbn* mutants compared to the control, and instead carotenes and other carotenoids preferentially accumulated in membrane and crystal fractions. In *fbn*7a, reduction in the proportion of β‐carotene and lycopene content in the membrane was observed (Figure 4b). Altered deposition of α‐tocopherol and PC‐8 across sub‐chromoplast compartments was also observed (Figure 4b). Accordingly, the relative composition of PG fractions greatly changed compared to the control when multiple *Sl*FBN were perturbed (Figure 4c). For example, in comparison to the single mutant *fbn*7a and its control, the proportion of α‐tocopherol and PC‐8 was reduced in the PG fractions but increased in the membranes. Together, these results suggest a reduced capacity of isoprenoid sequestration in the PG caused by multiple *Sl*FBN disruptions.

### Proteome analysis of *fbn* mutants exposes abnormal protein targeting to PG surface

To investigate the direct changes caused by lack of functional PG‐related FBNs on PGs, proteomic analysis was performed on fruit sub‐plastidial chromoplast fractions, focusing on PG‐enriched fractions. The composition of tomato fruit PG proteome was defined based on plastid proteins consistently identified in control and azygous samples, with potential contaminants filtered out according to subcellular localisation data (Table S7). From the *Sl*FBN family, four members, *Sl*FBN1, ‐2a, ‐4 and ‐8, were readily identified as PG‐associated proteins (Figure 4d). Surprisingly, *Sl*FBN7a was not detected in either the PG fraction or further membrane‐related fractions. Other PG‐associated proteins (Lundquist et al., 2012) including NAD(P)H‐ubiquinone oxidoreductase C1 (NDC1), tocopherol cyclase (VTE1), PYP/PES1, plastoglobular protein of 18 kD (PG18) and flavin‐reductase related (FRR) were detected. Moreover, the vesicle‐inducing protein in plastids 1 (VIPP1) was found. This protein is known for its roles in the biogenesis and repair of thylakoid membrane protein complexes and conferring tolerance to membrane stress (Theis et al., 2019), and therefore considered a putative PG‐associated protein.

Both in triple and quadruple *fbn* mutants, target *Sl*FBN1, ‐2a, and ‐4 were absent in all analysed fractions. Indeed, only a few PG representatives were detected in *fbn* mutant proteomes. This included FRR, a PG unknown for the triple and LOXB for the quadruple mutants. Thus, PG protein composition is influenced by mechanisms that require functional FBNs.

### *Sl*FBN7a modulates esterification capability in flower chromoplasts

**Figure 5:**
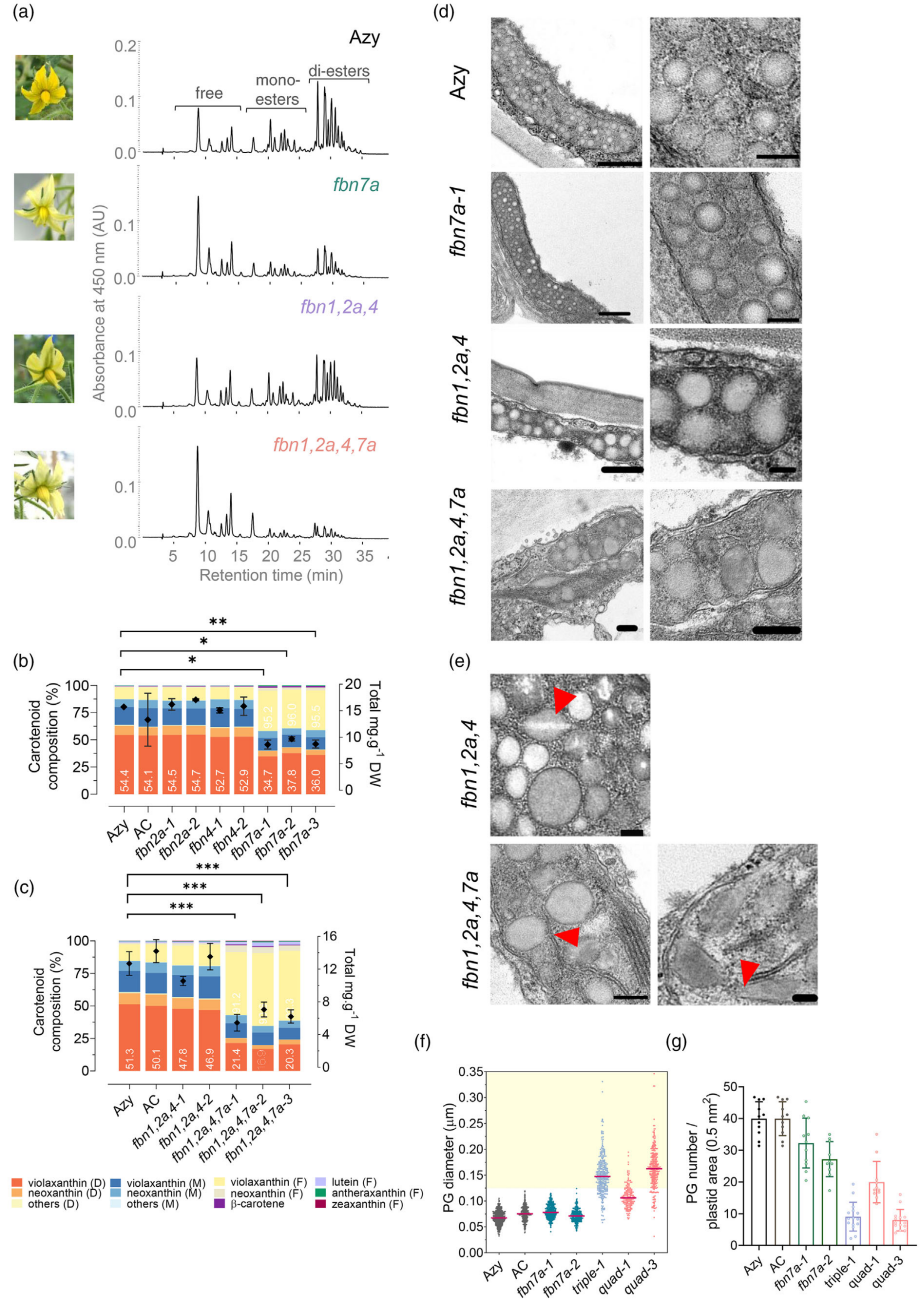
*Sl*FBN7a regulates carotenoid esterification driven colour retention in tomato flowers, while *Sl*FBN1, ‐2a, 4 affect plastoglobuli (PG) morphology. (a) HPLC chromatogram traces for control (Azy), *fbn7a*, triple (*fbn1,2a,4*) and quadruple (*fbn1,2a,4,7a*) mutants. Peaks corresponding to carotenoids in free forms, mono‐ and di‐esters are indicated. Carotenoid composition and total levels of single (b) and multiple (c) *fbn* mutants. Data (*n* ≥ 4, biological replicates) were analysed by one‐way anova with Dunnett's post‐tests; asterisks denote statistically significant differences (*P < 0.05, **P < 0.01, ***P < 0.001) compared to azygous (Azy) control. Error bars indicate ±SD. D, di‐esters; F, free forms; M, mono‐esters. (d) Representative transmission electron (TEM) micrographs of flower chromoplasts from Azy and *fbn* mutants. Detailed view of PG is shown on the right panels. Scale bars = 500 nm (left panels), and 50 nm (right panels). (e) Irregular shapes assumed by aberrant triple and quadruple PGs. Red head arrows indicate PG features found in mutants as crystalloid‐like structure, physical continuity with thylakoid remnants and irregular PG morphology. (f) Scatter plots showing diameter of PGs found in TEM petal micrographs. Yellow area highlights supersized PGs. (g) PG number per chromoplast area.

The yellow pigmentation of tomato flowers is determined by xanthophylls in their esterified form conjugated to medium‐length fatty acids (C14:0–C16:0), forming mono‐ and di‐esters (Ariizumi et al., 2014; Neuman et al., 2014). While *fbn*2a and *fbn*4 were typically bright yellow, *fbn7*a petals appeared pale (Figure 5a). Quadruple mutants also failed to develop yellow pigmentation, showing a more severe phenotype than *fbn*7a. Triple *fbn* mutant flowers only exhibited an apparently less intense yellow colour. To better understand the biochemical basis of the pale phenotype, detailed compositional analysis of carotenoids in petal extracts was performed using high‐performance liquid chromatography (HPLC‐PDA) to resolve carotenoids in free, mono‐ and di‐ester forms (Figure 5a–c; Table S5). Carotenoid levels and composition significantly changed in *fbn7a* petals. The total levels were decreased by ~50% relative to the control. The free carotenoids became predominant in the *fbn7a* compositional profile (threefold increase), followed by a decrease in the proportion of mono‐ and di‐esters (~70 and 65% of the values found in control, respectively) (Figure 5b). No significant changes in content or the composition of *fbn*2a and *fbn*4 petals were detected. Analysis of high‐order *fbn* mutants further confirmed the major role of *Sl*FBN7a in modulating the esterification capability of flower chromoplasts. Pale yellow petals of *fbn1,2a,4,7a* were similar to *fbn*7a and primarily accumulated free xanthophylls (>4‐fold higher versus control) while having lower levels of esterified xanthophylls. The decrease in mono‐ and di‐ester proportions (45 and 30% of the values found in control) exceeded those observed in *fbn*7a solely (Table S5), suggesting cumulative disruption in the esterification capability when multiple *Sl*FBNs are defective. Importantly, *fbn1,2a,4,7a* flowers had noticeable β‐carotene quantities, which are often analytically masked by xanthophyll esters in non‐saponified extracts (Price et al., 2018). Meanwhile, triple mutants (e.g. *fbn1, 2a, 4*), which had an unedited FBN7a protein, retained their quantitative and qualitative carotenoid content, supporting the FBN7a role in the regulation of esterification activity in flower chromoplasts.

To explore potential compensatory molecular mechanisms triggered by the targeted loss of *Sl*FBNs, the expression of other *Slfbn*s encoding PG‐associated proteins in flowers was compared (Figure S8). In petals, lower mRNA levels of the target *Slfbn*7a, highly expressed in flowers, were found in *fbn7a* and quadruple *fbn1,2a,4,7a* mutants. Importantly, transcript levels of *Slfbn*7b and *Slfbn*8 displayed no significant perturbation in *fbn* mutants, suggesting a lack of compensatory transcriptional response. Moreover, expression of the genes encoding PG‐located biosynthetic enzymes, including *Slccd*4B and *Slpes*1/*pyp* remained largely unchanged, except for a low level decrease of *Slpes*1 mRNA levels found in *fbn7a* mutants. This latter gene transcript encodes the acyltransferase responsible for xanthophyll esterification in tomato flowers (Ariizumi et al., 2014). Expression of *psy*1, the gene encoding the first enzyme of the carotenoid pathway, also remained unchanged in *fbn* mutants. These results suggested a lack of major transcriptional compensatory mechanisms triggered by the loss of functional *Sl*FBNs.

A similar compositional profile was also confirmed in the extra T_1_ *fbn* mutants (Figure S4; Table S5), although in this case, the total carotenoid content of triple (*Cas* ^+^ */fbn1,2a,4*) mutants was significantly reduced compared to wild‐type Ailsa Craig (AC), which may reflect the absence of azygous comparators at this stage. Furthermore, the total carotenoid amounts found in triple (*Cas* ^+^/*fbn1,2a,4*) and quadruple (*Cas* ^+^/*fbn1,2a,4,7a*) mutants were equivalent, indeed lower than those found in the double (*Cas* ^+^/*fbn2a,4*) mutant, supporting *Sl*FBN1/CHRC as one of the main contributors to xanthophyll sequestration in flower chromoplasts.

### FBNs are required for PG formation and chromoplast development

To investigate the effect of PG‐targeted *Sl*FBN deficiency on PG formation and chromoplast architecture, petals and fruit pericarps were examined under transmission electron microscopy (TEM). The plastids in petals were present in different forms (Ariizumi et al., 2014), ranging from chloroplast‐like structure (CLS) (early stage) to the fully re‐differentiated carotenoid‐rich chromoplasts. Early stage plastids had thylakoids organised in grana and low numbers of electron‐dense PGs (Figure 5d). Mature chromoplasts instead displayed the typical abundant small PGs (diameter ~60 nm), with a less electron‐dense core where carotenoids are sequestered. In pale yellow *fbn7a* petals, PGs were similar to the control in size and morphology but appeared less in number (Figure 5d–g). High‐order *fbn* mutants had chromoplasts with great variation in sub‐structures, including thylakoid remnants, with few and supersized PGs (diameter >140 nm; Figure 5f). In some cases, their abnormal PGs seemed to be associated with the persistent dismantled thylakoids and showed a discontinuous lipid monolayer instead of a smooth surface. Additionally, they displayed crystalloid‐like inclusions (e.g. lightly stained zones) in the form of filaments (Figure 5e). Particularly in quadruple mutants, with predominantly non‐esterified xanthophylls, PGs even assumed elongated to spindle‐shaped forms, instead of round structures, filled with homogeneous electron‐dense filaments. Interestingly, early stage plastids of high‐order *fbn* mutants exhibited a typical CLS with only a few supersized PGs (Figure S5).

**Figure 6:**
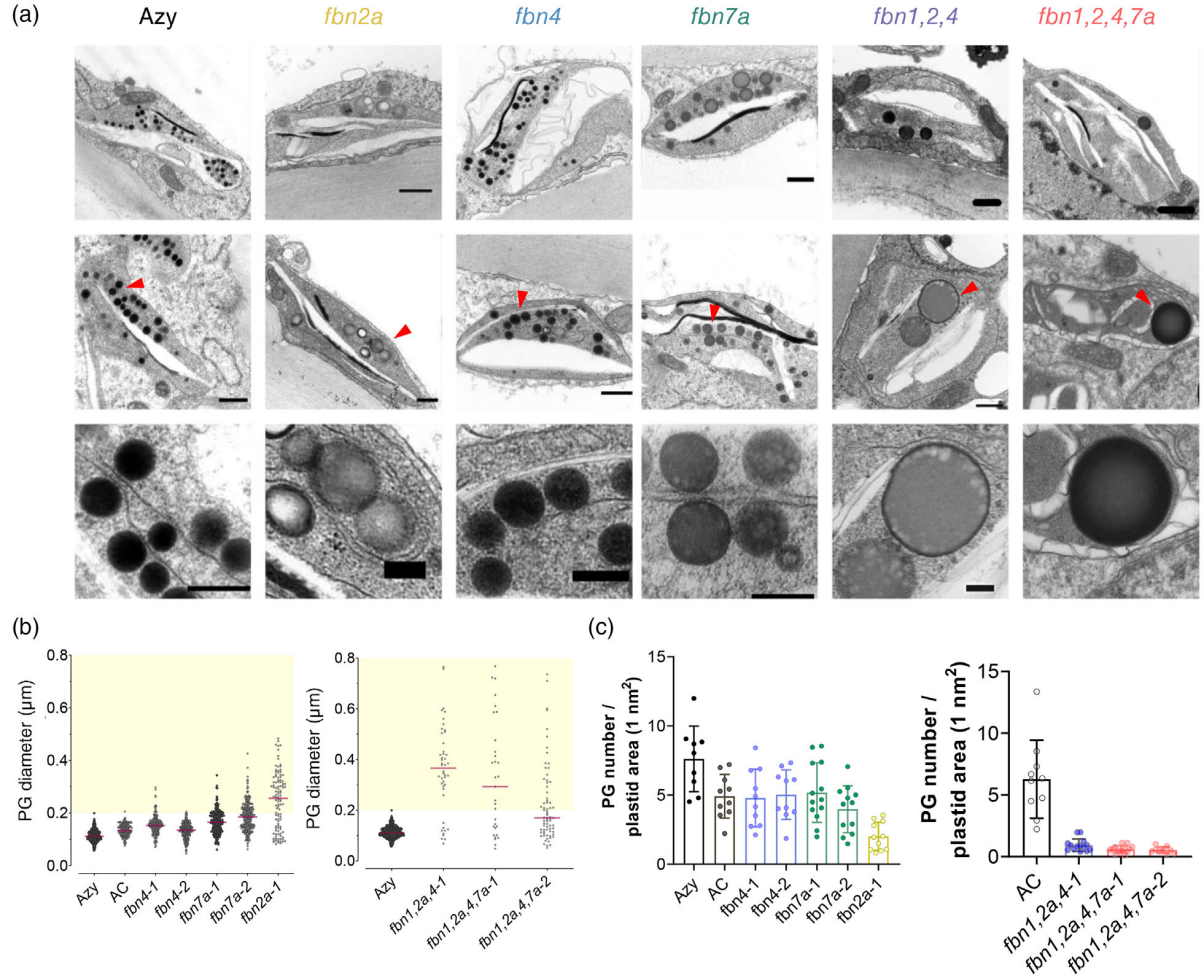
Altered fruit chromoplast ultrastructure of *fbn* mutants. (a) Two representative transmission electron (TEM) micrographs of fruit chromoplasts (5 days post‐breaker stage) from Azy and *fbn* mutants; scale bars = 0.5 μm. Red arrows indicate plastoglobulis (PGs) with a zoomed‐in view shown in the bottom panels; scale bars = 0.2 μm. Giant PG formation was observed in some chromoplasts of triple (*fbn1,2a,4*) and quadruple (*fbn1,2a,4,7a*) mutants. (b) Scatter plots showing the diameter of PGs found in TEM fruit micrographs. Yellow area highlights supersized PGs. (c) PG number per chromoplast area.

Unlike flowers, ripe fruits in tomato contain more diverse carotenoid‐sequestering structures including endomembranes, carotenoid crystals and bigger PGs (Nogueira et al., 2016). The chromoplasts found in control samples displayed crystals of lycopene and β‐carotene and several high‐electron‐dense PGs ~100 nm in diameter. PGs of single *fbn2a* and *fbn7a* mutants changed in number and morphology, whereas *fbn4* chromoplasts remained similar to control (Figure 6). In *fbn2a* mutants, supersized PGs (diameter >200 nm) were observed at high frequency, with some of them displaying electron‐lucent areas at their core. In *fbn7a* mutants, fruit PGs were found with a less electron‐dense core, and only a small proportion corresponded to supersized PGs (at least 25%). The changes in PG electron density are likely to be due to altered chemical composition, affecting extraction during preparation (Hempel et al., 2014).

Triple and quadruple mutants displayed significant alterations in fruit chromoplast structure consistent with their carotenoid profile. They frequently failed to develop typical globular PGs. Those PGs detected were found to be unusually big, with some even reaching giant size (diameter >500 nm). Lycopene crystals seemed to be less developed in comparison with the control, consistent with lower lycopene levels (Figure 6).

### Metabolome and lipidome alterations associated with FBN deficiency

The impact of PG‐associated FBNs on the plant metabolome focused on high‐order *fbn* mutants. Changes in the metabolic composition of primary metabolites were addressed by GC–MS profiling of polar and non‐polar‐saponified extracts of fruits and leaves (Table S6). In total, 117 metabolites were identified across the genotypes. Principal component analysis (PCA) using the combined dataset revealed little variation in the primary/intermediate metabolite composition of the *fbn* mutants compared to their respective controls. The exception was green fruits (MG), where a separation between *fbn* mutants and controls was attained (Figure S6). A limited number of metabolites were found to be altered in the *fbn* mutants compared to their azygous controls. In leaves, MG and ripe fruits, 18, 12, and 8 metabolite differentiators were detected, respectively, and included representative amino acids, sugars, organic acids, isoprenoids and fatty acids (Table S6). For example, *Sl*FBN deficiency led to unexpected changes in amino acid content of *fbn* fruits; these changes included increases in glutamine and threonine at the MG stage (Figure S6b) and significant decreases in phenylalanine in ripe fruits (Figure S6c). Among the fatty acids released through saponification, myristic acid (C14:0) decreased consistently in the *fbn* samples of ripe fruit (Figure 6c), while in leaves, other minor saturated species were also significantly altered, such as pentadecanoic acid (C15:0) and heptadecanoic acid (C17:0) (Figure S6d).

**Figure 7:**
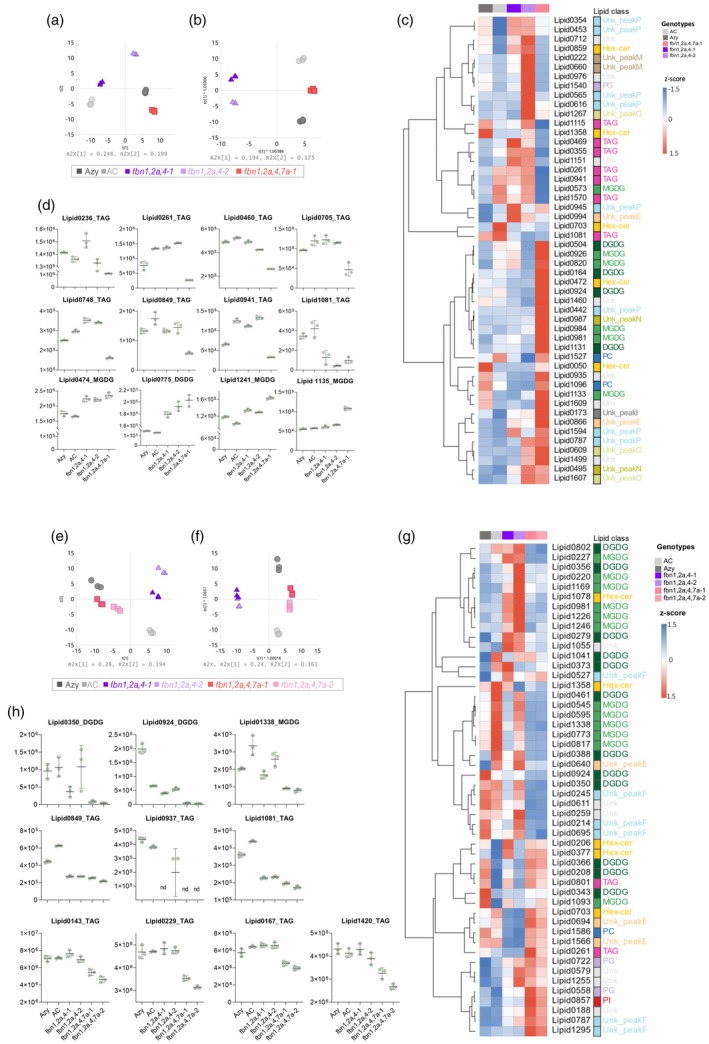
Untargeted lipidomic analysis of high‐order *fbn* mutants. Data was obtained from LC–MS based on the hydrophilic interaction liquid chromatography method. Panels (a–d) correspond to leaf tissue, and panels (e–h) correspond to flower tissue analysis. Score plots of principal component analysis (a, e) and orthogonal partial least squares discriminant analysis (OPLS‐DA) (b, f) based on untargeted LC–MS chemical features. Data were log‐transformed and Pareto scaled. Hierarchical clustering of the top 50 variables (c, g) with the highest VIP scores identified by OPLS‐DA. Heat maps are coloured by red and blue colours indicating higher and lower abundance, respectively, as measured by row standardised *z*‐scores derived from LC–MS peak intensity (*n* = 3 biological replicates). Genotypes are annotated as coloured boxes on the top‐right of the heatmap. The boxes on the right of the heatmap indicate the lipid classes. The graphs representing quantitative changes (peak area normalised by dry weight) (d, h) of selected chemical features based on VIP scores and statistical significance (anova followed by Fisher's LSD, *α* = 0.05).

PGs are known to be implicated in lipid remodelling (van Wijk & Kessler, 2017); therefore, a deeper lipidomic analysis was performed based on LC–MS: (i) an untargeted lipidome and (ii) a specific targeted analysis for triacylglycerol (TAG) and its derivatives. The score plot of the PCA from the untargeted lipidome illustrates the clustering of the *fbn* mutants and their controls (Figure 7a). Orthogonal partial least squares discriminant analysis was implemented to identify differences between the control, triple and quadruple *fbn* mutant lipidomes. The highest variable on projection (VIP) scores, which rank the importance of features in distinguishing genotypes, were retrieved. These combined analyses revealed *Sl*FBN deficiency had the strongest effects on plastidial membrane galactolipids (monogalactosyldiacylglycerol, MGDG; digalactosyldiacylglycerol; DGDG) and TAG; the latter presumably representing pools derived from the cytosol and plastids. In leaves, while several MGDG and DGDG‐related species increased in both triple and quadruple mutants, several TAG species were found depleted, particularly in quadruple mutants. The same trend for the TAG lipid class was found in petals, but in contrast to leaf tissues, MGDG and DGDG‐related lipid species featured among the top representatives decreased in *fbn* mutants. Targeted TAG analysis (Figure S7) allowed further confirmation of impaired esterification capability associated with defective *fbn7a* alleles; several TAG‐related species were found exclusively depleted in quadruple mutants either in leaves or flowers. Notably, lowered TAG‐related lipid species were found both in triple and quadruple mutants, suggesting that aberrant PG morphologies associated with *Sl*FBN deficiency *per se* are sufficient to trigger changes in lipid metabolism. Additionally, TAG‐specific *fbn1,2a,4,7a* changes imply that FBN7a may modulate broader lipid esterification, not only carotenoids.

## DISCUSSION

The present work has utilised the CRISPR/Cas‐based technology to systematically interrogate the functionality of the FBN multigene family. The repertoire of edited alleles generated (Figure 2; Table S3) both as individual and high‐order mutants has been confirmed by transcript and protein analysis. Collectively, this suite of mutants represents a valuable genetic resource (Figures 3 and 5). The *fbn* mutants have been phenotyped, isoprenoid composition determined, analysis of the plastid ultrastructure carried out and the metabolome and lipidome profiled. Overall, the comprehensive characterisation of the *fbn* mutants clearly indicated that FBNs are essential components of the PG, and although functional redundancy is evident with respect to isoprenoid biosynthesis, tissue‐specific roles linked to lipid storage or redox homeostasis‐related lipophilic compounds exist.

Cellular ultrastructure analysis revealed dramatic effects on PGs present in the *fbn* mutants. For example, deficiency in multiple *Sl*FBNs such as the combination of *Sl*FBN1, 2a, 4 resulted in heterogeneous and supersized PGs, while unaffected plastoglobular structures were found in individual *fbn* mutants (Figures 5 and 6; Figure S5). FBNs have been previously proposed to prevent PG coalescence (Rey et al., 2000) and promote PG formation via thylakoid budding. This hypothesis is supported by the present study where the high‐order *fbn* mutants presented dismantled thylakoids and discontinuous monolayers resulting in irregular PG structures (Figure 5d). The mechanism by which FBN proteins are targeted towards PGs and participate in their biogenesis and stability remains to be determined. Certain hypotheses suggest biophysical mechanisms driving the spatial partitioning (Olzmann & Carvalho, 2019), while other arguments favour post‐translational modifications influencing the sub‐organelle location (Lohscheider et al., 2016). However, a dedicated molecular machinery targeting and transporting FBN into PGs has not been described to date.

The cellular and spatial proteome analysis of isolated PG fractions performed in this work confirms the partitioning of PG‐localised *Sl*FBN1, ‐2a, ‐4 and ‐8, but mixed results were obtained for *Sl*FBN7a. The imaging of *Sl*FBN7a‐YFP fusion expression indicates co‐localisation with PG structures in leaves (Figure 1), but undetectable levels of trypsin‐derived peptides in fruit tissue were found, contrasting previous results reported in tomato (Szymanski et al., 2017). Technical differences (GEL‐LC versus in‐solution digestion) and/or analytical sensitivity limitations could be attributed, but also tissue‐specific implications may be involved since *Slfbn*7a transcript levels are (i) significantly higher in flower tissue than in fruit and (ii) different coloured flower phenotypes were observed in the collection of *fbn* mutants. The carotenoid analysis obtained here indicated that *Sl*FBN7a deficiency led to a substantial loss of carotenoid esters in petals. The enzyme catalysing carotenoid esterification in tomato flowers is PES1 (Ariizumi et al., 2014; Lewis et al., 2021), a non‐specific acyltransferase that is PG localised. However, the nature of the interaction between PES and FBN7a in tomato remains understudied. Candidate mechanisms have considered complex formation, as recently reported in rapeseed flowers (Li et al., 2023), or the assembly of enriched FBN‐substrate (carotenoid and lipids) condensates. Further hypotheses arise from the data collected in the present study. For example, the further decrease in carotenoid esters observed in the triple and quadruple mutants containing deficient *Sl*FBN7a suggests that a specific macromolecular configuration would be favourable for optimal activity, beyond bilateral interactions. In addition, the effect of *Sl*FBN7a on esterification activity is also extended to other lipid classes as several TAGs species were significantly decreased in both leaves and flowers of quadruple mutants carrying defective *fbn*7a alleles (Figures S7 and S8). Specific paralog functionality is also inferred from the data generated. For example, in the case of *Sl*FBN2a, the deficient mutants present higher levels of PC‐8. The *At*FBN2 has been shown to interact with NDC1 (Torres‐Romero et al., 2021). This enzyme reduces the oxidised pool of PQ‐9 in PG, thus regenerating the substrate (plastoquinol‐9, PQH2‐9) required by the tocopherol cyclase VTE1 to produce PC‐8 (Piller et al., 2011; Torres‐Romero et al., 2021). The *Sl*FBN2a could have a positive effect on PC‐8 accumulation in a similar manner, either by direct association with NDC1 or indirectly by facilitating access/exposure to substrate.

In both cases, *Sl*FBN7a and *Sl*FBN2a, it is suggested that the molecular mechanisms of FBNs may go beyond protein–protein interaction, and functional macromolecular assemblies are potentially involved. Biosynthetic metabolons and similar molecular scaffolds have been previously described for different biosynthetic pathways, including sectors of the isoprenoids family (Fraser, Schuch, et al., 2000; Wurtzel, 2019). The evidence from the *Sl*FBN2a and *Sl*FBN7a reported here, together with those of *At*FBN2 (Piller et al., 2011), *At*FBN5 (Kim et al., 2015), the algal chloroplast SEC14‐like protein (CPSFL1) (Hertle et al., 2020) or the tomato tocopherol binding protein (*Sl*TBP) (Bermúdez et al., 2018), suggests a putative role of FBNs as biosynthetic ancillary proteins that could bind and/or transfer substrates to enzymes directly or indirectly by operating as large macromolecular structures or biosynthetic hubs. The overall cellular and metabolic effect of the FBNs studied here is summarised in Figure 7 and a mechanistic model is proposed.

**Figure 8:**
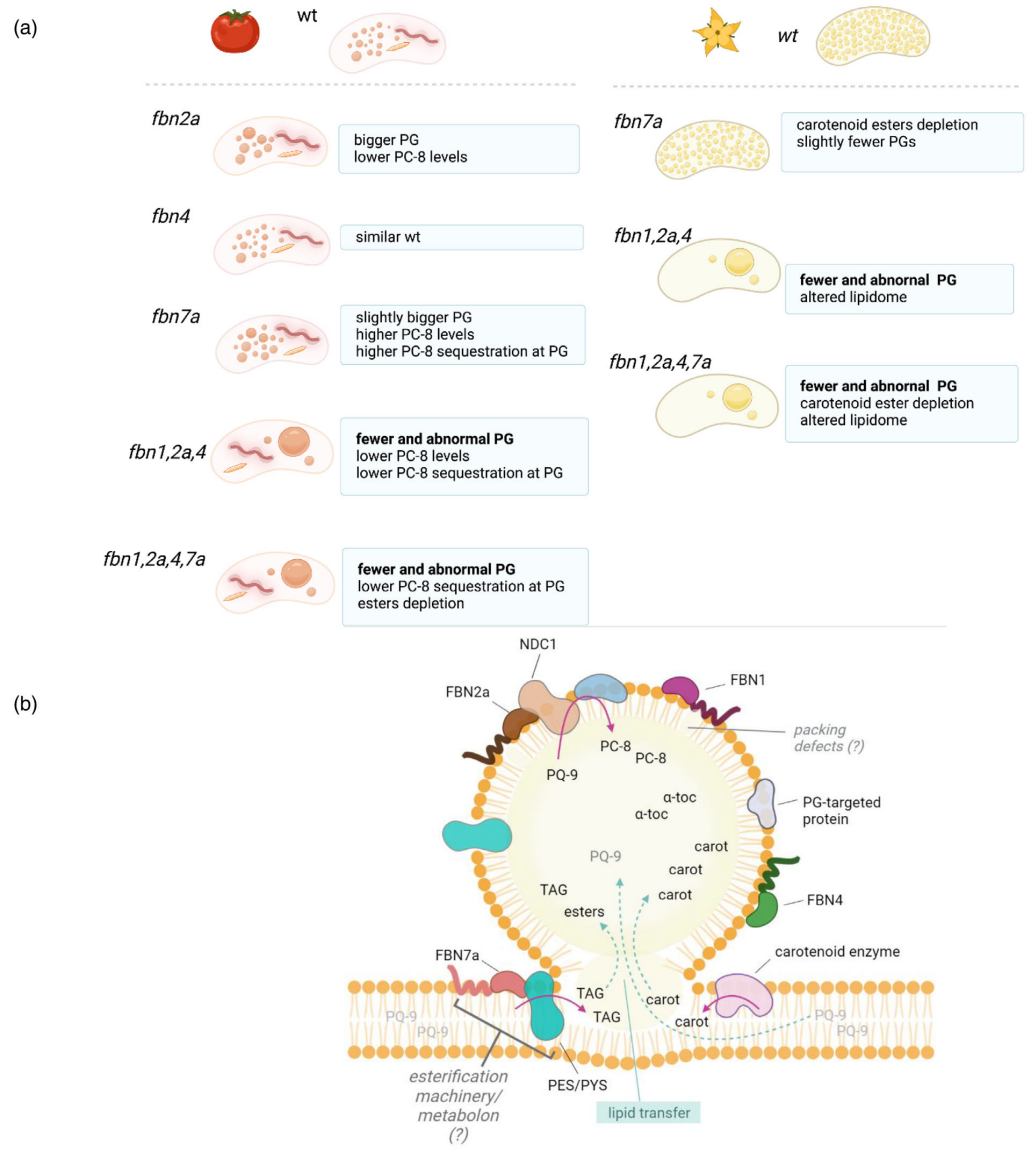
Summary of changes associated with *Sl*FBN deficiency and proposed model. (a) Knockout of *Sl*FBNs alters morphology and number of plastoglobuli (PGs), followed by changes in lipid metabolism and sequestration. Functional redundancies of *Sl*FBN were exposed by aberrant PG phenotypes found in high‐order *fbn* mutants. *Sl*FBN functional divergence, instead, was dissected through metabolite profiling of *fbn* mutants. Specifically, *Sl*FBN7a influences esterification capability having a key role in flower colour retention. *Sl*FBN2a together with *Sl*FBN7a, in turn, regulate PG‐related metabolite accumulation having opposite effects on PC‐8 levels. (b) Proposed model. As PG composition is affected by *Sl*FBN functionality, we propose that during PG formation and growth, their surface area may serve as docking sites for FBN proteins to bind and recruit enzymes that sustain PG growth and core lipid remodelling, forming complexes such as *Sl*FBN2a/NDC1 (based on previously shown interaction between FBN2 and NDC1, Torres‐Romero et al., 2021). Thus, *Sl*FBNs may shape PG lipid core via local isoprenoid biosynthesis. Alternatively, other members such as FBN7a might support PG formation as part of the biosynthetic machinery for ester production, where it may selectively facilitate the availability/transfer of the newly synthesised lipids by acyltransferases (TAG and/or carotenoid esters), from plastidial membranes to PG. In the absence of functional *Sl*FBN7a, transfer of other prenyl lipids such as PQ‐9 could be favoured, which in turn boosts PG‐local production of PC‐8. Collectively, we propose that *Sl*FBNs can shape PG composition not only by supporting PG local isoprenoid synthesis but also by influencing the lipid transfer from thylakoids to PG. VTE1, tocopherol cyclase.

Concomitant with ultrastructural, proteome, and isoprenoid profiling changes in the PG of the *fbn* mutants, pronounced effects on the global lipidome in different tissues (fruit, flower, leaf) also occur. Depletion of several fatty acids in ripe fruit and leaf (Figure S6), alterations in galactolipids and TAGs composition in triple and quadruple mutants (Figures S7 and S8) such as reciprocal levels of MGDG and DGDG between flower and leaf, illustrate the impact on the plant's lipidome. In particular, the effect in leaf and flowers on plastidial galactolipids and their turnover into TAGs (Higashi et al., 2018; Lippold et al., 2012), which appears compromised by alterations in the PG structure and its proteome. The differential metabolic demands between respective plastid types should also not be ignored. Interestingly, several examples in the scientific literature report alterations in metabolite composition and metabolic reprogramming in engineered outputs targeting isoprenoid production (Enfissi et al., 2019; Fraser et al., 2007; Nogueira et al., 2023), also accompanied by profound perturbations in plastid structure and transcriptome. However, the collection of *fbn* mutants studied here presented subtle global effects on the steady state metabolome or key related transcripts. Collectively, this would infer that changes in metabolite composition have a greater effect on orchestrating the adaptation of cellular structures (Figure 8).

In summary, multiplex gene editing tools have been used to decipher the functionality of the FBN multigene family. Functional redundancy is evident, but collectively FBNs play a major role in PG biogenesis and stability. In addition, the results reveal tissue‐specific roles of individual FBNs, for example, *Sl*FBN7a and *Sl*FBN2a in carotenoid and TAG esterification and PC‐8 formation, respectively. It must be emphasised, though, that PG alterations and related lipophilic compositional changes are more pronounced when multiple FBNs have been knocked out. The findings of the present work expand the knowledge on fundamental aspects of metabolic compartmentalisation and tissue specialisation in plant cells, emphasising the role of lipoprotein particles in plastid physiology. Considering the relevance of these sub‐plastid structures in plant development and adaptation to environmental cues (Coulon et al., 2024; van Wijk & Kessler, 2017), and the interactive/dynamic nature of the FBNs discussed above, genetic resources based on multiplexed mutants represent a valuable strategy for elucidating the role of FBNs and their individual and collective mechanisms of action.

## MATERIALS AND METHODS

### Phylogenetic analysis

For phylogenetic analysis, BlastP searches were performed using the protein sequences of *At*FBN (van Wijk & Kessler, 2017) as queries against the tomato genome (Solanaceae Genomic network, http://solgenomics.net). For pepper (*Capsicum annuum*, cv. Zunla), sequences were retrieved from the PepperHub database (Liu et al., 2017). The sequences were aligned using the MUSCLE package available in the MEGA X software with default parameters, and Neighbour‐Joining phylogenies with 1000 bootstrap replications were created with the distances calculated according to the best model indicated by MEGA (Kumar et al., 2018).

### Subcellular localisation and confocal microscopy

For *Sl*FBN subcellular localisation, the full‐length CDS of *Slfbn*2a, *Slfbn*4, *Slfbn*7a (without stop codon) were cloned into the p2YGW7, resulting in a C‐terminally tagged YFP protein. The PG marker used, *Atfbn*1a fused in‐frame with *cfp*, was from the vector p2CGW7 (Vidi et al., 2006). Arabidopsis mesophyll protoplasts were isolated as previously described (Wu et al., 2009). Protoplast transfection assay was performed using a polyethylene glycol‐based method (Yoo et al., 2007) immediately after the protoplast isolation. After 24 h, fluorescence emitted from the protoplasts was observed using a TCS‐SP8 laser scanning confocal microscope (Leica Microsystems CMS, Wetzlar, Germany) as follows: excitation 514 nm, emission 520–570 nm for YFP; excitation 458 nm, emission 465–505 nm for CFP; excitation 514 nm, emission 650–740 nm for chlorophyll autofluorescence. Emitted fluorescence was false‐coloured in green (YFP), red (CFP) and blue (chlorophyll). The confocal images shown are maximal projections of selected planes of a *z*‐stack.

### Plant material and growth conditions

Tomato plants (*Solanum lycopersicum*, cv. AC) were grown under greenhouse conditions with a 16/8‐h day night photoperiod at 25°C/19°C, respectively. For experiments, homozygous/biallelic and azygous segregants were grown at the same time in the same glasshouse room.

### *CRISPR‐Cas9* constructs for generating tomato *fbn* mutants

Genomic sequences 5′‐G(N)_19_NGG were selected to design two gRNA spacer sequence targeting specifically *Slfbn*1, *Slfbn*2a, *Slfbn*4, *Slfbn*7a, avoiding off‐target effects, using CRISPR‐P software (Lei et al., 2014). Secondary structure analysis of target‐sgRNA sequences was performed with the programme RNA folding tool (Hamada et al., 2016). The standardised modular cloning system Golden Gate MoClo was used (Engler et al., 2014; Weber et al., 2011). The Cas9 expression cassette consists of a 2x35S Cauliflower Mosaic Virus promoter (pICH51288) and a plant codon‐optimised Cas9 coding sequence, pcoCas9 (Fauser et al., 2014), which was domesticated for removal of internal BbsI and BsaI restriction sites, both parts used to assemble a Level 1 transcriptional unit (pICH47742::2x35S::Cas9) according to Lawrenson et al. (2015). For the gRNAs, a 5′‐tailed primer harbouring the individual *Slfbn* spacer (guide sequence), BsaI recognition site and compatible vector overhang was used to generate specific gRNA amplicons by PCR from a gRNA scaffold template (AddGene no. 46966; for primers; see Table S9). Each gRNA PCR fragment was individually assembled with Arabidopsis U6 small RNA promoter (pICSL01009::AtU6p, AddGene no. 46968) in the appropriate Level 1 vector (plCH47751, plCH47761, plCH47772, plCH47781, pICH47791, pICH47732, pICH47742, pICH47751) depending on the position of each gRNA in the final binary vector as described by Lawrenson et al. (2015). A neomycin phosphotransferase II (NPTII) cassette (pICH47732::NOSp::NPTII, Addgene no. 51144) was used as a selectable marker for plant transformation. For single *fbn* mutants, NOSp::NPTII; 2x35Sp:Cas9; Level 1 AtU6p::gRNA1, Level 1 AtU6p::gRNA2 were assembled into the final Level 2‐based binary vector. For building multiplex *Slfbn* gRNA vectors, NOSp::NPTII; 2x35Sp:Cas9; Level 1 AtU6p::gRNA1 to AtU6p::gRNA(n) were assembled into Level P vector and produced multiplex vectors P1‐M1 (designed for targeting *Slfbn*1, *Slfbn*2a, *Slfbn*4) and P1‐M2 (designed for targeting *Slfbn*1, *Slfbn*2a, *Slfbn*4, *Slfbn*7a). Restriction‐ligation reactions were performed in 20 μl volume in a thermocycler for 40 cycles of 37°C for 3 min followed by 16°C for 4 min, with a final incubation of 5 min at 50°C and 5 min at 80°C. All assembled plasmids were validated by restriction enzyme analysis and confirmed by Sanger sequencing. Final binary vectors were transformed into *Agrobacterium tumefaciens* (strain LBA4404).

### *Agrobacterium tumefaciens*‐mediated transformation and selection of transformants

*Agrobacterium tumefaciens*‐mediated transformations of the *S. lycopersicum* cv AC were performed as previously described in Pino et al. (2010) using cotyledon segments from 8‐day‐old seedlings precultured with *Agrobacterium* containing the CRISPR/Cas9 constructs of interest. Selective regeneration medium containing kanamycin (100 mg L^−1^) was used for explant selection. After 8 weeks, well‐rooted T_0_ plantlets were acclimatised to greenhouse conditions and their leaves sampled for genotyping.

### Detection and determination of targeted mutagenesis

Genomic DNA was purified from leaves using the DNeasy 96 plant kit (Qiagen, Venlo, the Netherlands) following the manufacturer's instructions. The presence/absence of the transgene in regenerated primary plants (T_0_) and offspring (T_1_ and T_2_ plants) was detected by PCR in the genomic DNA using NPTII and Cas9‐specific primers (Table S9). To characterise the Cas9‐induced mutations in the transformed plantlets, specific primers for *Slfbn*, whose binding positions are about 250 bp upstream of the target site, were used for PCR amplification. Amplicons were analysed by Sanger sequencing. For those with multiple peaks in chromatograms, the PCR product was cloned into TOPO‐TA (Thermo Fisher Scientific, Waltham, MA, USA) and, at least five clones per sample were sequenced. T_1_ progeny of the confirmed T_0_ gene‐edited lines were PCR‐genotyped for screening of inherited mutant alleles (either in homozygous or biallelic state) and Cas9 null segregants. The azygous segregant line descended from an original CRISPR‐single and from a CRISPR‐multiplex vector transformant was selected as a control line. Phenotyping was performed on T_2_ Cas9 null segregant homozygous offspring for *fbn* singles, triple and quadruple mutants, unless stated otherwise. For multiplex CRISPR constructs, an extra set of gene‐edited lines for triple (*fbn*1,2a,4), quadruple (*fbn*1,2a,4,7a) as well as double mutant (*fbn2a,4*), the latter generated through a P1‐M1 transformant whose *Slfbn*1 edition failed, was obtained in AC background lacking green shoulder (glk2/uniform recessive). Their corresponding T_1_ progeny harboured Cas9‐gRNA expression cassette Cas^+^.

### Isoprenoid determination and quantification

Isoprenoids (carotenoids, tocochromanols and chlorophylls) were extracted from lyophilised tissue powder (15 mg) as described by Enfissi et al. (2010). From homogenised (powdered) freeze‐dried tomato material, isoprenoids (carotenoids, chlorophylls, quinones and tocopherols) were extracted using methanol (250 ml) and chloroform (500 ml) with 50 mM Tris–HCl pH 7.5 (250 ml) added subsequently to aid the formation of a liquid: liquid partition. The resulting hypophase after centrifugation at 3000 × ***g*** for 3 min was removed and the suspension re‐extracted with chloroform. The pooled chloroform extract was dried under nitrogen gas and resuspended in ethyl acetate (50 ml) for HPLC separation. HPLC analysis was carried out with a Waters Alliance system; the separation column used was a C30 bonded silica‐based reverse‐phase column 250 × 4.6 mm i.d. 5 μm (YMC [Lancaster, UK], Thermo Fisher Scientific, Phenomenex [Macclesfield, UK]) and corresponding C30 guard column (20 × 4.6 mm, 5 μm). Mobile phase solvents used were (A) methanol, (B) methanol:water (80:20) (vol.) containing 0.2% (w/vol.) of ammonium acetate and (C) methyl‐tert butyl ether. A flow rate of 1 ml min^−1^ was used and the gradient ran over 60 min. At 0–6 min the mobile phase composition is 95% A, 5% B; at 7 min: 80% A, 5% B, 15% C; 12 min: 80% A, 5% B, 15% C; 32 min: 30% A, 5% B, 65% C; 54 min: 30% A, 5% B, 65% C; 56 min: 95% A, 5% B; 62 min: 95% A, 5% B. An injection volume of 10 μl is used and the column oven is maintained at 25°C. The elute is monitored continuously across the wavelength range of 200–600 nm using an on‐line PDA (Fraser et al., 2000).

A similar extraction of isoprenoids was carried out for UPLC with an online PDA. Compounds were analysed by reverse‐phase chromatography using a UPLC system (Acquity, Waters Company, Wilmslow, UK) equipped with a PDA detector (Acquity, Waters Company). A UPLC BEH‐C18 column (100 mm × 2.1 mm; 1.7 μm, Acquity, Waters Company) was used for separation as described in Nogueira et al. (2013). In both cases, HPLC and UPLC peak identification was achieved by comparison of characteristic UV/Vis spectrum with authentic standards, reference spectra and retention times (Fraser et al., 2007). For xanthophyll esters in flowers, a spectra table is available in Table S10. Quantification was performed using dose–response curves obtained from authentic standards when available.

### Sub‐chromoplast fractionation

Chromoplasts were isolated from fruits (90 g) at B3–B4 stage, and sub‐compartments were fractionated using a discontinuous gradient of sucrose, according to Nogueira et al. (2013). In brief, ripening tomato fruit (150 g) were deseeded and cut into pieces (ca 1 cm squares), then soaked in extraction buffer 50 mM Tris–HCl, 1 mM DTT, 1 mM EDTA with 0.4 m sucrose and adjusted to pH 7.8. Homogenisation was performed with a Waring blender (2 × 3 sec blasts). The slurry was filtered through 2–4 layers of muslin. The suspension was centrifuged at 5000 × ***g*** for 10 min at 4°C. The resulting pellet was resuspended in extraction buffer and centrifuged at 9000 × ***g*** for 10 min at 4°C. The crude plastid pellet was resuspended in 50 mM Tricine pH 7.9, containing 2 mM EDTA and 2 mM DTT with 5 mM sodium bisulphite. Lysis of the plastids was carried out with a Potter‐Elvehjem tissue lyser. The lysed fraction was separated on a discontinuous gradient with 38, 20 and 5% w/v sucrose present in the buffer used to resuspend plastids.

### qPCR expression analyses

Total RNA was extracted from flowers and fruit pericarps using the RNeasy kit (Qiagen) according to the manufacturer's instructions. RNA quality was assessed by agarose gel electrophoresis. Total RNA (1 μg) was treated with DNaseI and converted into cDNA using the QuantiTect Reverse Transcription kit (Qiagen), according to the manufacturer's protocols. Real‐time qPCR assays were performed in duplicate using the RotorGene SYBR green PCR kit (Qiagen) on the Rotor‐Gene Q, with approximately 10 ng of reverse‐transcribed RNA. Primer sequences are listed in Table S1. Relative expression was calculated as described by Quadrana et al. (2013). For reference gene selection, expression stability of four known reference genes—*cac, exp, act*1 and *act*2 (Cheng et al., 2017; Expósito‐Rodríguez et al., 2008), were evaluated using GeNorm (Vandesompele et al., 2002) with *act*2 and *cac* selected based on the lowest expression stability values (M) of 0.362 and 0.438, respectively.

### Transmission electron microscopy

Segments from pericarp fruit and mature, opened flowers were fixed at room temperature in solution (3% [v/v] glutaraldehyde, 4% [v/v] formaldehyde buffered with 0.1 m PIPES buffer pH 7.2) and then stored at 4°C for at least 24 h until processing. Samples were post‐fixed in buffered 1% (w/v) osmium tetroxide and uranyl acetate, washed, dehydrated in a graded series of acetone and embedded in resin. Ultrathin sections were stained with Reynolds lead citrate and imaged on a Tecnai T12 Transmission Electron Microscope (Field Electron and Ion Company, Hillsboro, OR, USA). Measurements were made in ImageJ v.2.0.0‐rc‐68/1.52e (National Institutes of Health, Bethesda, MD, USA).

### Immunoblotting and proteomic analysis

For immunoblotting, ripe fruit homogenates (150 mg) were extracted with extraction buffer containing 0.1 m Tris‐Cl (pH 7.5–8.0), 6 m urea, 2 m thiourea, 0.2% (v/v) Triton X‐100, 0.2% (w/v) sarcosyl, and 2 mM DTT. Total protein content was quantified using the Bradford method (Bradford, 1976), and 10 μg of total protein was separated on a 12.5% w/v polyacrylamide gel and blotted to PVDF membrane. Blots were probed for the presence of the FBN1 using a polyclonal antibody raised against *Capsicum* FBN1/PAP (Deruere et al., 1994) and analysed using the method described by Fraser et al. (1994).

For proteomic analysis, total protein was extracted from isolated fractions, run in the SDS‐PAGE gel, subsequently digested with trypsin and analysed into nano LC–MS/MS as described by Nogueira et al. (2013). Analyses were conducted in an AdvanceBio Peptide Map column (2.1 × 100 mm, 2.7 μm, Agilent Technologies Inc., Santa Clara, CA, USA) and Infinity II 1290 UHPLC coupled to a 6550 iFunnel QTof (Agilent Technologies Inc.). Data analyses were performed using Spectrum Mill MS Proteomics Workbench (Rev B.06.00.201, Agilent Technologies Inc.) and Mascot Distiller (v2.4.2.0, Matrix Science, London, UK) as described in Nogueira et al. (2013).

### Metabolite profiling by GC–MS

Polar and non‐polar saponified extracts from fruits and leaves were prepared and analysed as previously described in Almeida et al. (2021). For polar and non‐polar metabolite analysis, freeze‐dried tomato tissue (10 mg) was extracted with methanol (400 μl); then, ultrapure water was added (400 μl). The suspension was mixed by rotary inversion for 1 h. Chloroform (800 μl) was then added, and the mixture was centrifuged for 5 min at 20 000 × ***g***. From the polar extract (upper phase) a 10 μl aliquot was removed, and D4‐Succinic acid (10 μl of a 0.1 mg ml^−1^ stock). From the non‐polar (lower phase), 700 μl was transferred and D27‐Myristic acid (10 μl) was added. The polar and non‐polar extracts were dried. Derivatisationwas performed using methoxyamine hydrochloride (MEOX) solution (1 mg ml^−1^) in pyridine; 30 μl of the MEOX solution was added to the dried polar or non‐polar extracts. Following heating for 1 h at 40°C, N‐methyl‐N‐trimethylsilyl (MSTFA; 70 μl) was added for a further 2 h at 40°C. Using 1 ml injections, GC/MS separations were conducted using a temperature gradient starting at 70°C for 1 min, followed by an 8°C min^−1^ increase to 325°C, holding the final temperature for 2 min. Data analysis was carried out using AMDIS (version 2.73), while confirmation of metabolites can be achieved through analysis of authentic standards or a database (e.g. NIST11, Bethesda, MD, USA; http://chemdata.nist.gov/mass‐spc/ms‐search/).

### Lipid analysis by liquid chromatography (LC)–MS/MS

Leaf, fruit and flower petals (5–10 mg) were extracted with chloroform: methanol (1 ml, 2:1, v/v). Analysis was performed as previously described by Drapal et al. (2022) for phospho‐ and galactosyl lipids and neutral lipids. Samples were processed with Agilent Profinder (v10.0 SP1, Agilent Technologies Inc.) and identification was performed through comparison to authentic standards and MS/MS fragmentation pattern.

### Statistical analyses

Univariate statistical analysis for comparison between control and *fbn* mutants was performed by Student's *t*‐test or anova followed by a *post hoc* test (as stated in figure and tables) with the level of significance set to 0.05, using GraphPad Prism 8 software. For multivariate statistical analysis, MetaboAnalyst 4.0 (Chong et al., 2019) and Simca^®^17 (Sartorius, Gottingen, Germany) software were utilised.

## CONFLICT OF INTEREST

The authors declare that they have no known competing financial interests or personal relationships that could have appeared to influence the work reported in this paper.

## AUTHOR CONTRIBUTIONS

Conceptualisation: JA and PDF; methodology and experimentation: JA, KL, LP‐F, MD and PDF; formal analysis: JA; investigation: JA; resources: PDF; data curation: JA; writing—original draft: JA; writing—review and editing: JA, LP‐F, MD, KL, EMAE and PDF; visualisation: JA; project administration: PDF and EMAE; funding acquisition: PDF and EMAE.

## Supporting information


**Figure S1.** Expression profile of FIBRILLIN (FBN) loci in pepper. Heatmap representation of *Cafbn* expression profile in pepper. Expression data were obtained from PepperHub (http://www.hnivr.org/), represented as FPKM‐normalised values.
**Figure S2.** CRISPR/Cas9 constructs for target gene editing of *Slfbn* genes. Scheme of final binary vectors containing the NPTII selectable marker, Cas9 driven by the 2x35S promoter and transcriptional units for gRNA expression (under control of U6 promoter). LB, left border; RB, right border.
**Figure S3.** Plant morphology of multiple knockout *fbn* mutants. (a) Representative fully expanded leaves (cv. Ailsa Craig) of 10‐weeks‐old Azy, AC and triple and quadruple *fbn* mutants. All plants were cultivated together in the same growth chamber. Orange arrows indicate lesions caused by fungal pathogens. Bar = 5 cm. (b) Internode length of the four equivalent internodes. (c) Maximum quantum efficiency of Photosystem II (Fv/Fm). Data (*n* > 5, biological replicates) are means and error bars indicate standard deviation (SD). Statistically significant differences between Azy and *fbn* mutant are indicated by *P‐*values (one‐way anova with Dunnett's post‐tests, *P* < 0.05).
**Figure S4.** Flower carotenoid composition of *Cas* ^+^ */Slfbn*‐edited lines. T_1_ *Slfbn*‐edited lines profiling by HPLC‐PDA. Carotenoid composition and total levels of double (*Cas*+/*fbn2a,4*), triple (*Cas*+/*fbn1,2a,4*) and quadruple (*Cas*+/*fbn1,2a,4,7a*) *fbn* mutants. Data (*n* = 3, biological replicates) were analysed by one‐way anova with Dunnett's post‐test; asterisks denote statistically significant differences (*P < 0.05, **P < 0.01, ***P < 0.001) compared to AC control. Error bars indicate ±SD. D, di‐esters; F, free forms; M, mono‐esters.
**Figure S5.** Aberrant plastoglobuli (PG) morphology found in flower plastids of high‐order *fbn* mutants. Representative transmission electron micrographs (TEM) of early stage plastids (bearing thylakoids organised in grana and a few electron dense PGs) from petals from Azy and *fbn* mutants. Detailed view of PGs was shown on the inset panels. Giant PG formation was observed in some plastids of quadruple (*fbn1,2a,4,7a*) mutants. Scale bars = 500 nm (main panels), and 50 nm (inset).
**Figure S6.** Effect of *Sl*FBN deficiency on tomato primary metabolism. (a) Scores plot obtained by principal component analysis (PCA) for metabolite levels measured in polar and non‐polar extracts in fruits at mature green (MG) and ripe (B7) stage, and in leaves (from top to bottom). Changes in metabolites associated with *Sl*FBN deficiency in MG (b), ripe fruits (c) and leaves (d). Quantification was determined relative to the internal standard and values are presented as mean ± SD from four biological replicates. Only significant changes compared to respective Azy control are shown (pair‐wise *t*‐test corrected for multiple comparison using Holm–Sidak's post‐test; Adjusted *P < 0.05, **P < 0.01, ***P < 0.001). Full data set available in Table S6. C14:0, myristic acid; C15:0, pentadecanoic acid; C17:0, heptadecanoic acid.
**Figure S7.** Targeted lipidomic analysis of triacylglycerol (TAG) and its derivatives. (a, c) Heat map of top 100 features identified by anova (*Post hoc* Fisher's LSD, *α* = 0.05, corrected by multiple comparisons) in leaf and flower, respectively. Red and blue colours indicate higher and lower abundance, respectively, as measured by column standardised *z*‐scores derived from LC–MS peak intensity (*n* = 3 biological replicates). Genotypes are indicated on the right of the heatmap. The boxes on the top of the heatmap indicate the lipid classes, and whether the variable is found among the highest VIP scores (top 100) identified by OPLS‐DA. (b, d) Selected chemical features from heat maps of leaf and petal, respectively, that show statistically significant changes on *fbn* mutants (anova followed by Fisher's LSD, *α* = 0.05).
**Figure S8.** Relative expression of *Slfbns* and other genes encoding metabolic enzymes associated with PG by qPCR. Values are expression levels normalised to *cac* and *act2* reference genes (mean ± SEM; *n* ≥ 3 biological replicates). Significant differences (Student's *t‐*test, *P < 0.05, **P < 0.01, ***P < 0.001) between *fbn* mutants and control are indicated.

**Table S1.** gRNA recognition sequences in *Slfbn* genes and off‐targets.
**Table S2.** Number of plants recovered from *Agrobacterium*‐mediated transformation harbouring mutations in the *Slfbn* target gene.
**Table S3.** *Slfbn* edited lines phenotyped in this study.
**Table S4.** Isoprenoid profile of fruits and leaves from *fbn* mutants determined by UPLC‐PDA.
**Table S5.** Isoprenoid profile of petals from *fbn* mutants determined by HPLC‐PDA.
**Table S6.** Metabolite levels measured in fruits (MG and B7 stage) and leaves by GC–MS.
**Table S7.** Proteome associated with PG fraction of tomato fruits from control and *fbn* mutant genotypes.
**Table S8.** Proteome associated with membrane fraction of tomato fruits from control and *fbn* mutant genotypes.
**Table S9.** Primers used in this study.
**Table S10.** Peak identification of isoprenoids analysed by HPLC‐PDA.

## Data Availability

Raw data files for lipidomics and proteomics are available through Mendeley data (DOI: 10.17632/9xmnybzgnd.1 and DOI: 10.17632/3hxz7c77r5.1). All other data generated or analysed during this study are included in this article (Appendix S1).
