# Supplementary material for: The FIBRILLIN multigene family in tomato, their roles in plastoglobuli structure and metabolism
Source: Plant J. 2025 Sep 9;123(5):e70447. doi: 10.1111/tpj.70447 (PMC12419240; doi:10.1111/tpj.70447)
Supplement: Supplementary file 1 — Figure S1. Expression profile of FIBRILLIN (FBN) loci in pepper. Heatmap representation of Cafbn expression profile in pepper. Expression data were obtained from PepperHub (http://www.hnivr.org/), represented as FPKM‐normalised values. Figure S2. CRISPR/Cas9 constructs for target gene editing of Slfbn genes. Scheme of final binary vectors containing the NPTII selectable marker, Cas9 driven by the 2x35S promoter and transcriptional units for gRNA expression (under control of U6 promoter). LB, left border; RB, right border. Figure S3. Plant morphology of multiple knockout fbn mutants. (a) Representative fully expanded leaves (cv. Ailsa Craig) of 10‐weeks‐old Azy, AC and triple and quadruple fbn mutants. All plants were cultivated together in the same growth chamber. Orange arrows indicate lesions caused by fungal pathogens. Bar = 5 cm. (b) Internode length of the four equivalent internodes. (c) Maximum quantum efficiency of Photosystem II (Fv/Fm). Data (n > 5, biological replicates) are means and error bars indicate standard deviation (SD). Statistically significant differences between Azy and fbn mutant are indicated by P‐values (one‐way anova with Dunnett's post‐tests, P < 0.05). Figure S4. Flower carotenoid composition of Cas + /Slfbn‐edited lines. T1 Slfbn‐edited lines profiling by HPLC‐PDA. Carotenoid composition and total levels of double (Cas+/fbn2a,4), triple (Cas+/fbn1,2a,4) and quadruple (Cas+/fbn1,2a,4,7a) fbn mutants. Data (n = 3, biological replicates) were analysed by one‐way anova with Dunnett's post‐test; asterisks denote statistically significant differences (*P < 0.05, **P < 0.01, ***P < 0.001) compared to AC control. Error bars indicate ±SD. D, di‐esters; F, free forms; M, mono‐esters. Figure S5. Aberrant plastoglobuli (PG) morphology found in flower plastids of high‐order fbn mutants. Representative transmission electron micrographs (TEM) of early stage plastids (bearing thylakoids organised in grana and a few electron dense PGs) from petals from A [file TPJ-123-0-s001.pptx]

## Slide 1
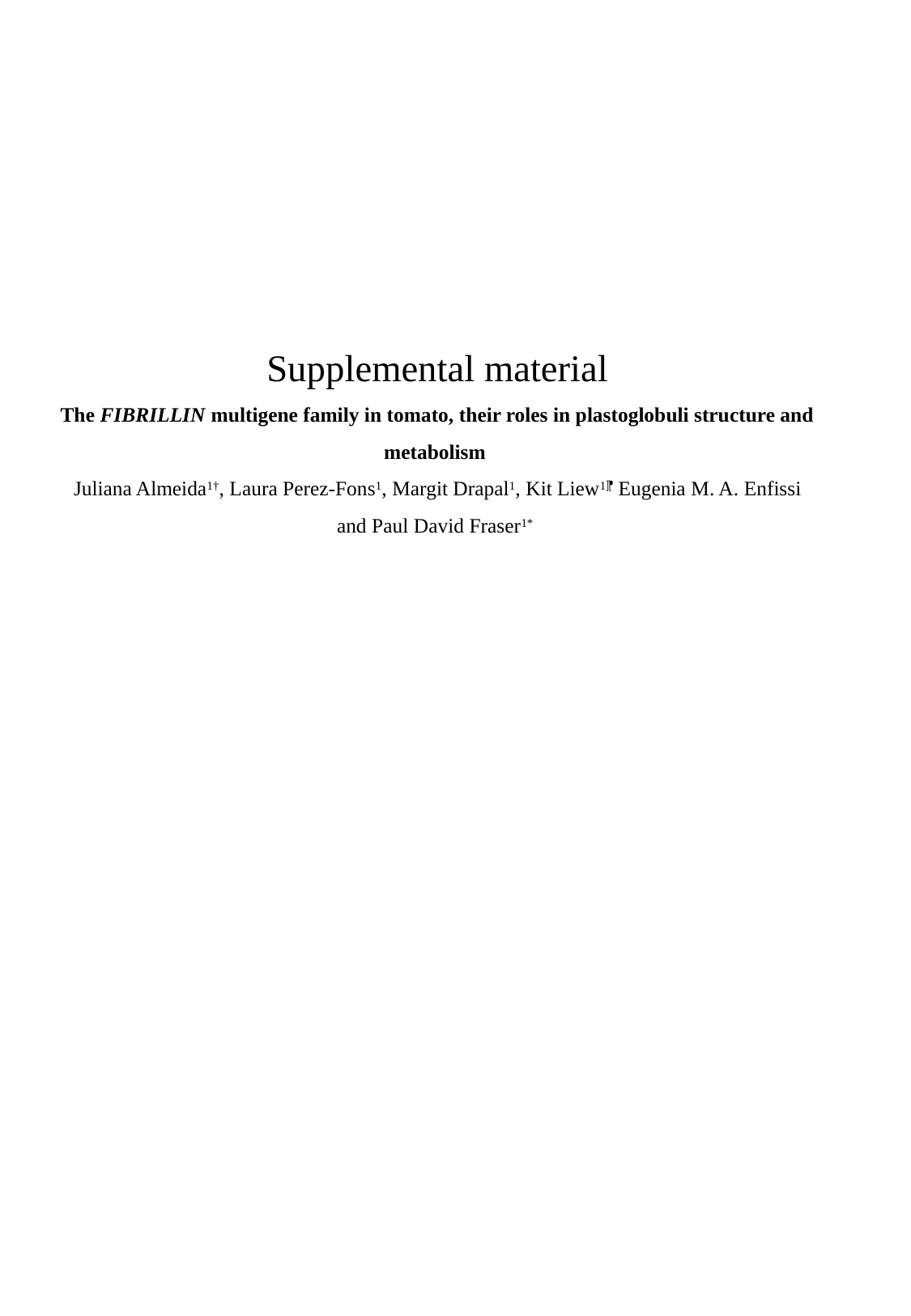

# Supplemental material The FIBRILLIN multigene family in tomato, their roles in plastoglobuli structure and metabolism Juliana Almeida1†, Laura Perez-Fons1, Margit Drapal1, Kit Liew1⁋ Eugenia M. A. Enfissi and Paul David Fraser1*

## Slide 2
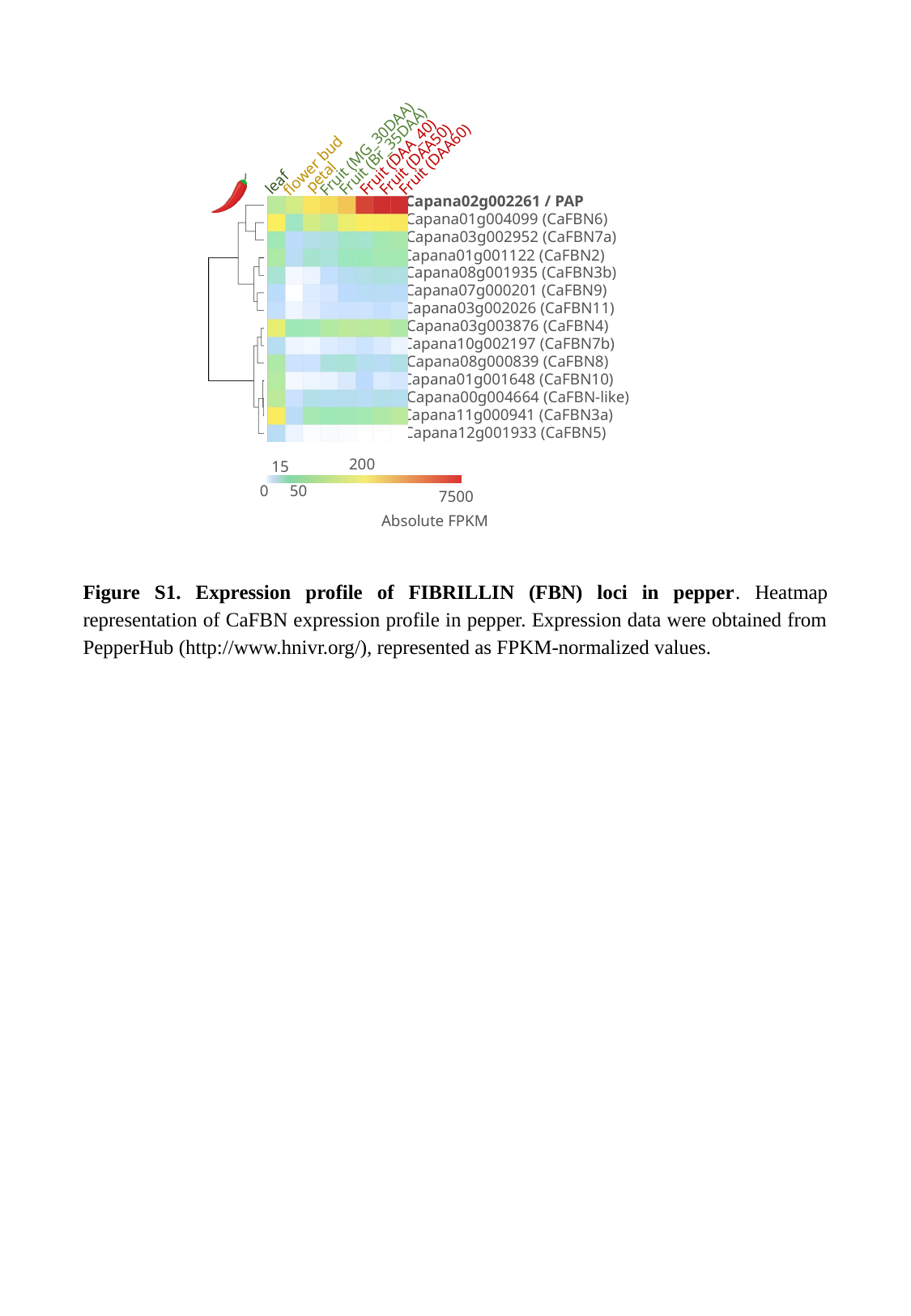

Fruit (MG_30DAA)
Fruit (Br_35DAA)
Fruit (DAA_40)
Fruit (DAA50)
Fruit (DAA60)
flower bud
petal
leaf
Capana02g002261 / PAP
Capana01g004099 (CaFBN6)
Capana03g002952 (CaFBN7a)
Capana01g001122 (CaFBN2)
Capana08g001935 (CaFBN3b)
Capana07g000201 (CaFBN9)
Capana03g002026 (CaFBN11)
Capana03g003876 (CaFBN4)
Capana10g002197 (CaFBN7b)
Capana08g000839 (CaFBN8)
Capana01g001648 (CaFBN10)
Capana00g004664 (CaFBN-like)
Capana11g000941 (CaFBN3a)
Capana12g001933 (CaFBN5)
200
15
0
50
7500
Absolute FPKM
Figure S1. Expression profile of FIBRILLIN (FBN) loci in pepper. Heatmap representation of CaFBN expression profile in pepper. Expression data were obtained from PepperHub (http://www.hnivr.org/), represented as FPKM-normalized values.

## Slide 3
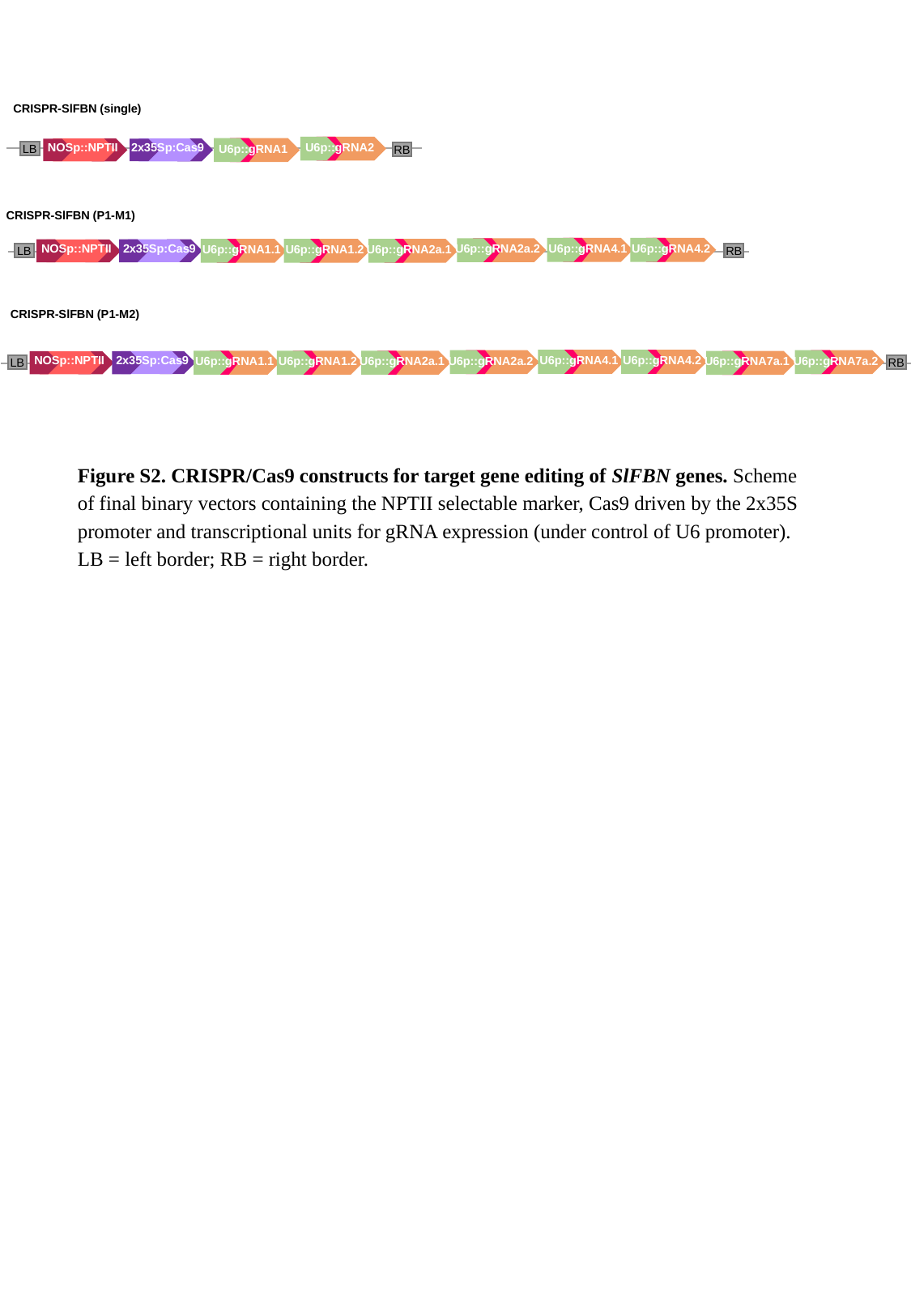

CRISPR-SlFBN (single)
U6p::gRNA2
NOSp::NPTII
2x35Sp:Cas9
U6p::gRNA1
LB
RB
CRISPR-SlFBN (P1-M1)
U6p::gRNA4.1
U6p::gRNA4.2
NOSp::NPTII
2x35Sp:Cas9
U6p::gRNA2a.2
U6p::gRNA1.1
U6p::gRNA1.2
U6p::gRNA2a.1
LB
RB
CRISPR-SlFBN (P1-M2)
U6p::gRNA4.1
U6p::gRNA4.2
NOSp::NPTII
2x35Sp:Cas9
U6p::gRNA2a.2
U6p::gRNA7a.2
U6p::gRNA1.1
U6p::gRNA1.2
U6p::gRNA2a.1
U6p::gRNA7a.1
LB
RB
Figure S2. CRISPR/Cas9 constructs for target gene editing of SlFBN genes. Scheme of final binary vectors containing the NPTII selectable marker, Cas9 driven by the 2x35S promoter and transcriptional units for gRNA expression (under control of U6 promoter). LB = left border; RB = right border.

## Slide 4
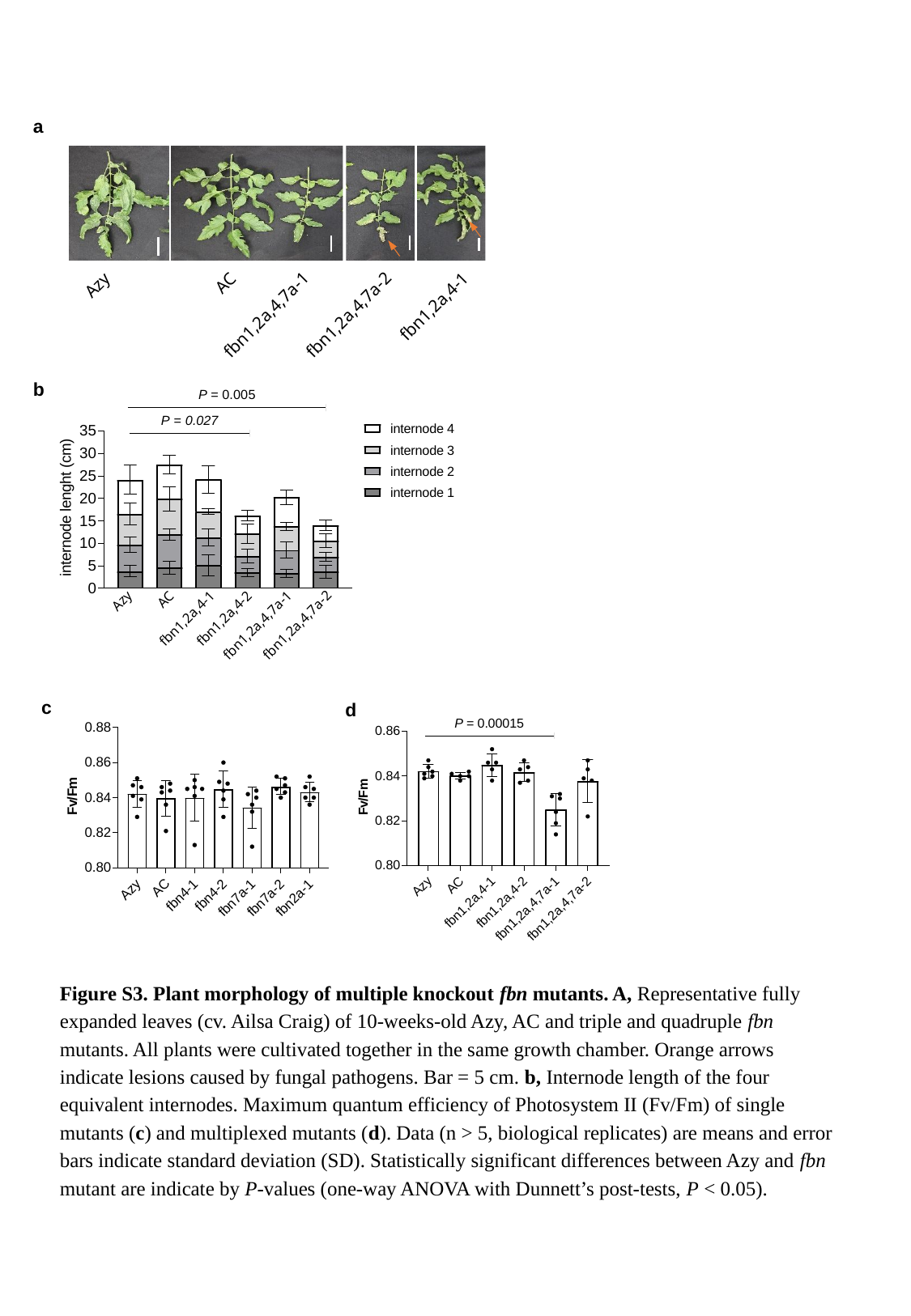

a
AC
Azy
fbn1,2a,4-1
fbn1,2a,4,7a-1
fbn1,2a,4,7a-2
b
AC
Azy
fbn1,2a,4-1
fbn1,2a,4-2
fbn1,2a,4,7a-1
fbn1,2a,4,7a-2
c
d
Figure S3. Plant morphology of multiple knockout fbn mutants. A, Representative fully expanded leaves (cv. Ailsa Craig) of 10-weeks-old Azy, AC and triple and quadruple fbn mutants. All plants were cultivated together in the same growth chamber. Orange arrows indicate lesions caused by fungal pathogens. Bar = 5 cm. b, Internode length of the four equivalent internodes. Maximum quantum efficiency of Photosystem II (Fv/Fm) of single mutants (c) and multiplexed mutants (d). Data (n > 5, biological replicates) are means and error bars indicate standard deviation (SD). Statistically significant differences between Azy and fbn mutant are indicate by P-values (one-way ANOVA with Dunnett’s post-tests, P < 0.05).

## Slide 5
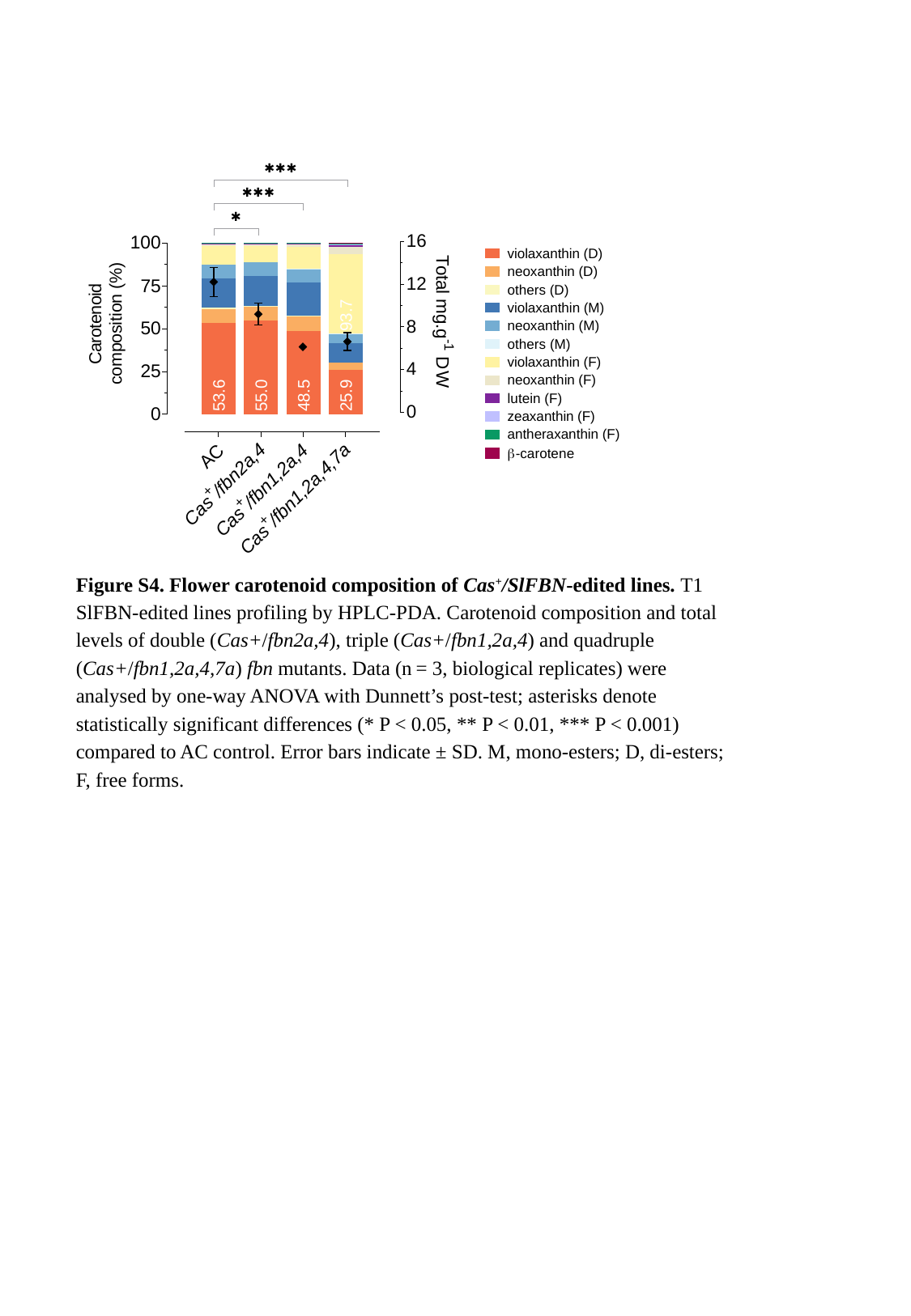

Figure S4. Flower carotenoid composition of Cas+/SlFBN-edited lines. T1 SlFBN-edited lines profiling by HPLC-PDA. Carotenoid composition and total levels of double (Cas+/fbn2a,4), triple (Cas+/fbn1,2a,4) and quadruple (Cas+/fbn1,2a,4,7a) fbn mutants. Data (n = 3, biological replicates) were analysed by one-way ANOVA with Dunnett’s post-test; asterisks denote statistically significant differences (* P < 0.05, ** P < 0.01, *** P < 0.001) compared to AC control. Error bars indicate ± SD. M, mono-esters; D, di-esters; F, free forms.

## Slide 6
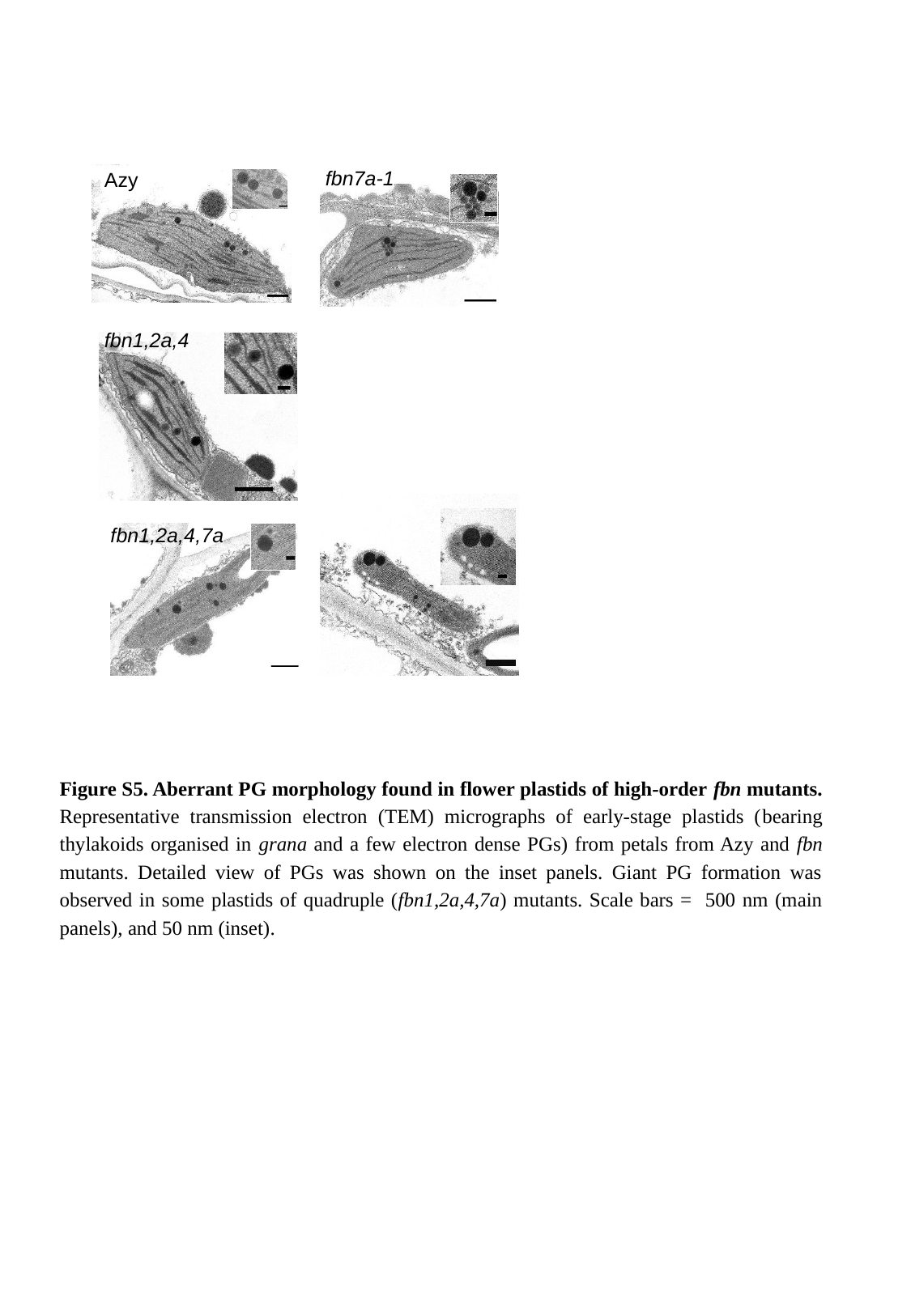

fbn7a-1
Azy
fbn1,2a,4
fbn1,2a,4,7a
Figure S5. Aberrant PG morphology found in flower plastids of high-order fbn mutants. Representative transmission electron (TEM) micrographs of early-stage plastids (bearing thylakoids organised in grana and a few electron dense PGs) from petals from Azy and fbn mutants. Detailed view of PGs was shown on the inset panels. Giant PG formation was observed in some plastids of quadruple (fbn1,2a,4,7a) mutants. Scale bars = 500 nm (main panels), and 50 nm (inset).

## Slide 7
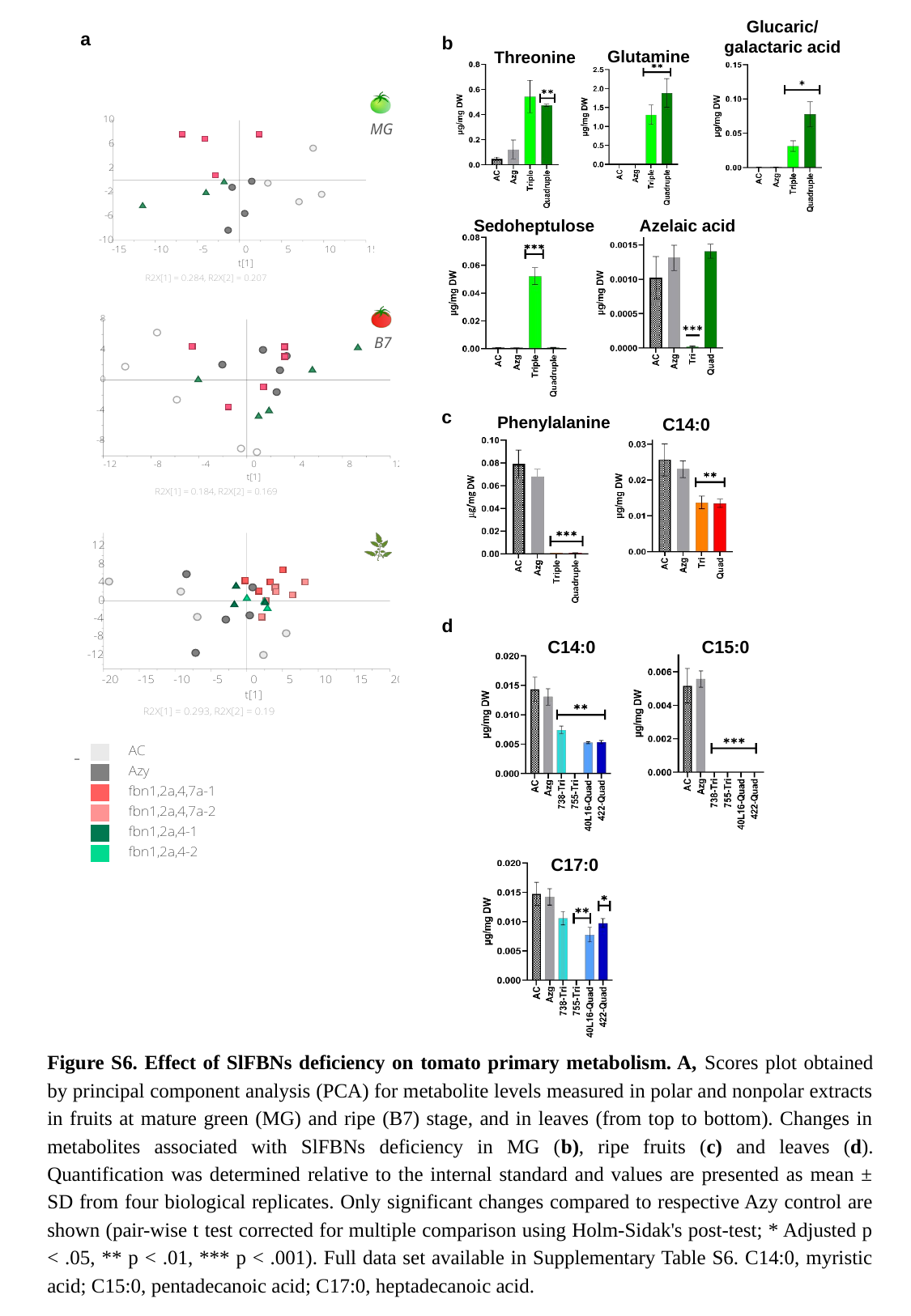

Glucaric/
galactaric acid
a
b
Glutamine
Threonine
MG
Sedoheptulose
Azelaic acid
B7
c
Phenylalanine
C14:0
d
C14:0
C15:0
C17:0
Figure S6. Effect of SlFBNs deficiency on tomato primary metabolism. A, Scores plot obtained by principal component analysis (PCA) for metabolite levels measured in polar and nonpolar extracts in fruits at mature green (MG) and ripe (B7) stage, and in leaves (from top to bottom). Changes in metabolites associated with SlFBNs deficiency in MG (b), ripe fruits (c) and leaves (d). Quantification was determined relative to the internal standard and values are presented as mean ± SD from four biological replicates. Only significant changes compared to respective Azy control are shown (pair-wise t test corrected for multiple comparison using Holm-Sidak's post-test; * Adjusted p < .05, ** p < .01, *** p < .001). Full data set available in Supplementary Table S6. C14:0, myristic acid; C15:0, pentadecanoic acid; C17:0, heptadecanoic acid.

## Slide 8
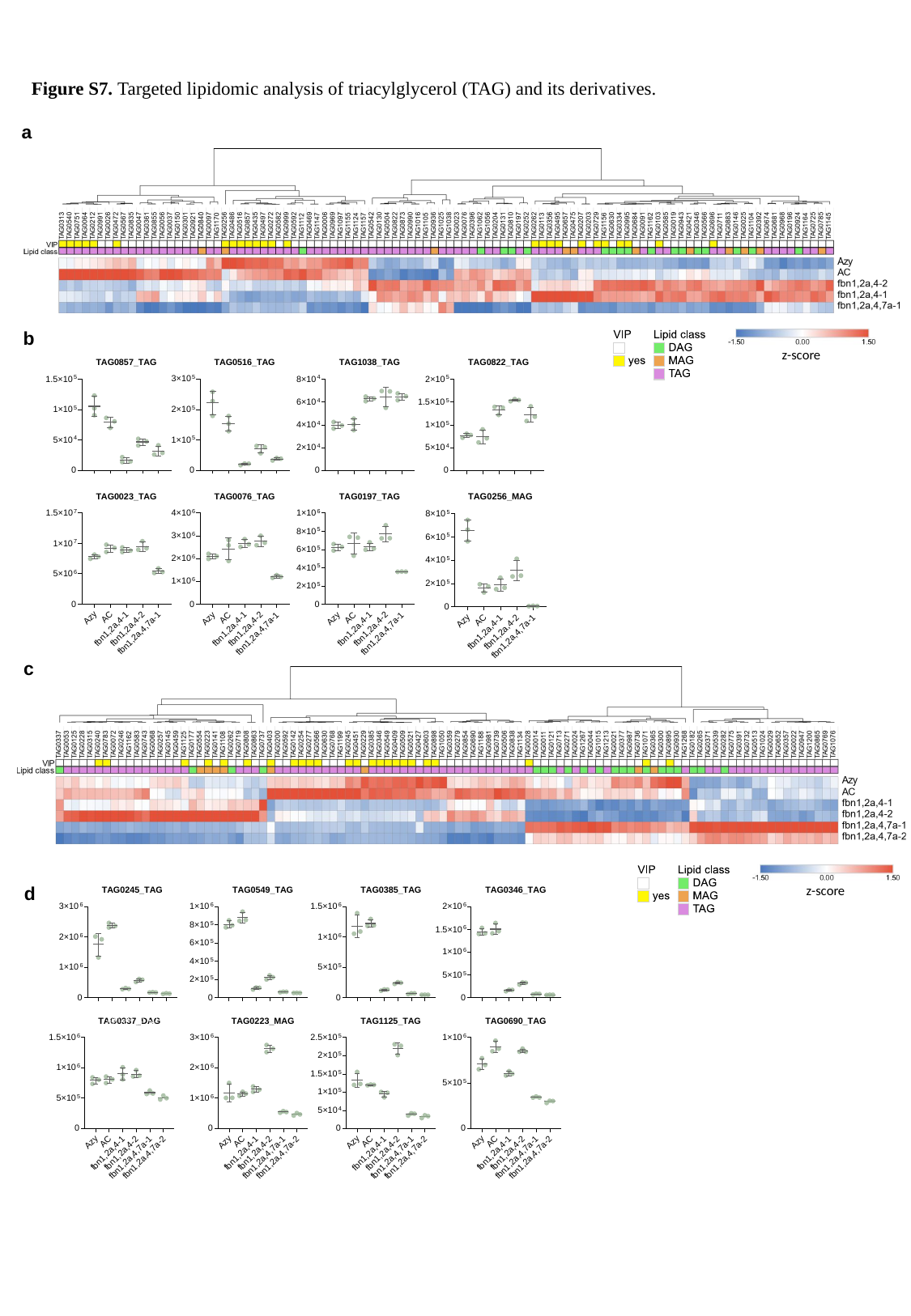

Figure S7. Targeted lipidomic analysis of triacylglycerol (TAG) and its derivatives.
a
b
z-score
c
d
z-score

## Slide 9
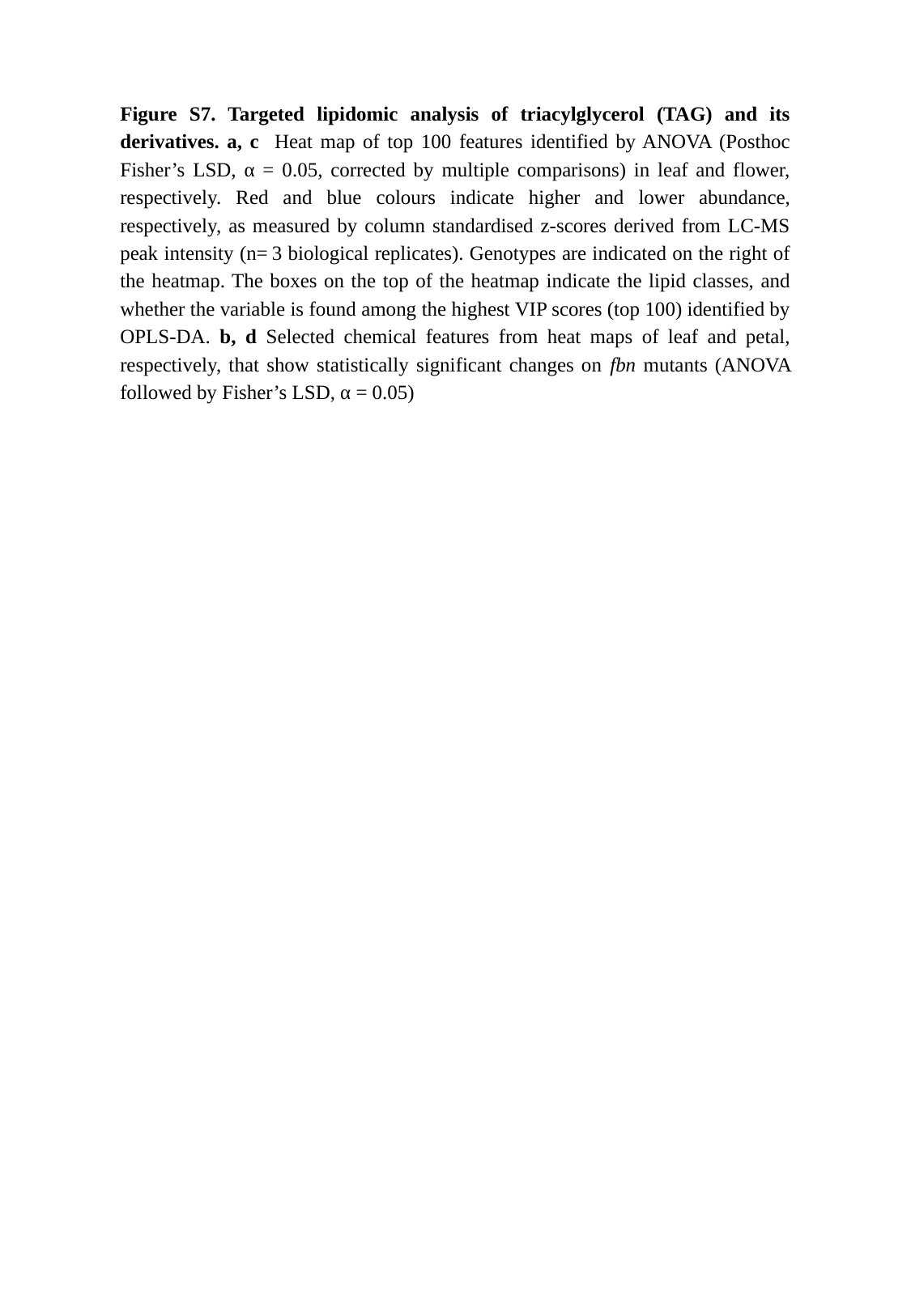

Figure S7. Targeted lipidomic analysis of triacylglycerol (TAG) and its derivatives. a, c Heat map of top 100 features identified by ANOVA (Posthoc Fisher’s LSD, α = 0.05, corrected by multiple comparisons) in leaf and flower, respectively. Red and blue colours indicate higher and lower abundance, respectively, as measured by column standardised z-scores derived from LC-MS peak intensity (n= 3 biological replicates). Genotypes are indicated on the right of the heatmap. The boxes on the top of the heatmap indicate the lipid classes, and whether the variable is found among the highest VIP scores (top 100) identified by OPLS-DA. b, d Selected chemical features from heat maps of leaf and petal, respectively, that show statistically significant changes on fbn mutants (ANOVA followed by Fisher’s LSD, α = 0.05)

## Slide 10
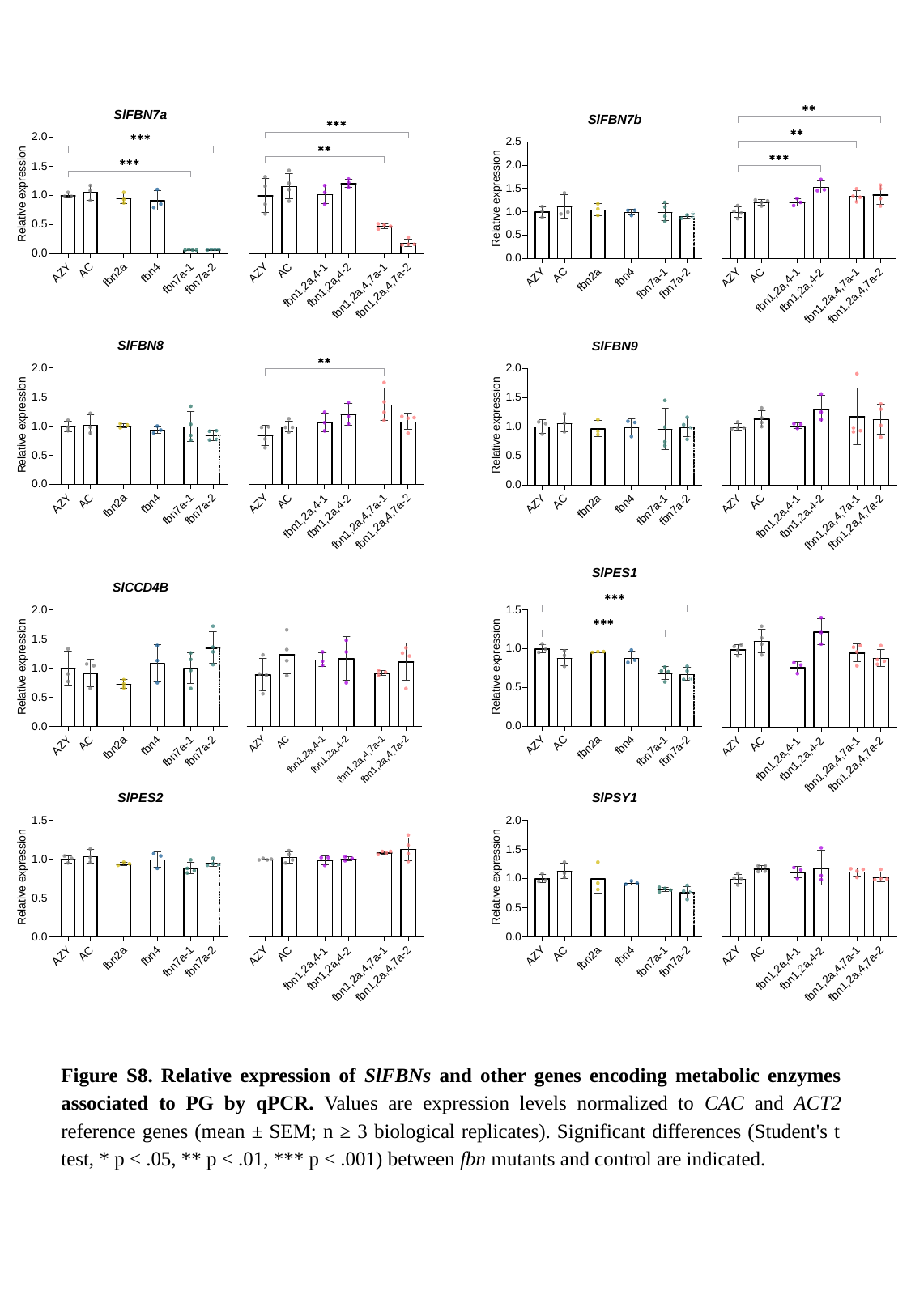

Figure S8. Relative expression of SlFBNs and other genes encoding metabolic enzymes associated to PG by qPCR. Values are expression levels normalized to CAC and ACT2 reference genes (mean ± SEM; n ≥ 3 biological replicates). Significant differences (Student's t test, * p < .05, ** p < .01, *** p < .001) between fbn mutants and control are indicated.
